# Discovery of Natural Kv1.3 Channel Inhibitors From Furanocoumarins of *Angelica dahurica* via Integrative Computational Screening and Electrophysiological Validation

**DOI:** 10.1002/cbdv.71923

**Published:** 2026-11-14

**Authors:** Yujie Chen, Yueshi Sha, Xianya Zhao, Binglin Lei, Liyang Guan, Nabeel Khalid Bhutta, Fan Zhang, Bingnan Han

**Affiliations:** ^1^ Zhejiang Provincial Engineering Research Center of New Technologies and Applications for Targeted Therapy of Major Diseases, Laboratory of Anti‐allergy Functional Molecules, College of Life Sciences and Medicine Zhejiang Sci‐Tech University Hangzhou China; ^2^ State Key Laboratory of Natural Medicines, School of Traditional Chinese Pharmacy China Pharmaceutical University Nanjing China

**Keywords:** *Angelica dahurica*, furanocoumarin, Kv1.3 channel inhibitor, molecular docking, molecular dynamics

## Abstract

The voltage‐gated potassium channel Kv1.3 regulates T‐cell activation and is a therapeutic target for autoimmune and inflammatory diseases. Although synthetic inhibitors such as PAP‐1 show potent activity, natural‐product alternatives remain underexplored. *Angelica dahurica* is rich in furanocoumarins and has anti‐inflammatory effects, thus, we investigated its constituents as Kv1.3 inhibitors. A library of 43 *A. dahurica* furanocoumarins was screened using molecular docking and MM/GBSA calculations against Kv1.3 (PDB: 7SSX). Eight compounds, representing four matched C‐5/C‐8‐substituted pairs, were selected based on screening results and availability for electrophysiological validation. Whole‐cell patch‐clamp recordings in human embryonic kidney 293 (HEK‐293) cells showed that all eight compounds inhibited Kv1.3 currents, with IC_50_ values of 4.27–22.42 µM. Three of the four matched pairs showed greater inhibitory potency for the C‐5‐substituted analogue, suggesting a preference for C‐5 substitution. At 10 µM, cell viability remained above 80% for CPD‐1 (bergamottin), CPD‐3 (cnidilin), and CPD‐4 (phellopterin) in both L929 fibroblasts and RAW 264.7 macrophages. Furthermore, molecular dynamics simulations indicated stable binding of CPD‐1 and CPD‐3 to Kv1.3, supported by RMSD, RMSF, and protein–ligand interaction profiles. These findings identify CPD‐1 and CPD‐3 as promising Kv1.3 inhibitors and support further investigation of *A. dahurica* furanocoumarins as lead compounds.

## Graphical abstract

Integrated workflow for identifying natural Kv1.3 inhibitors from *Angelica dahurica* furanocoumarins through computational screening, compound isolation and identification, electrophysiological validation, and molecular dynamics simulations.

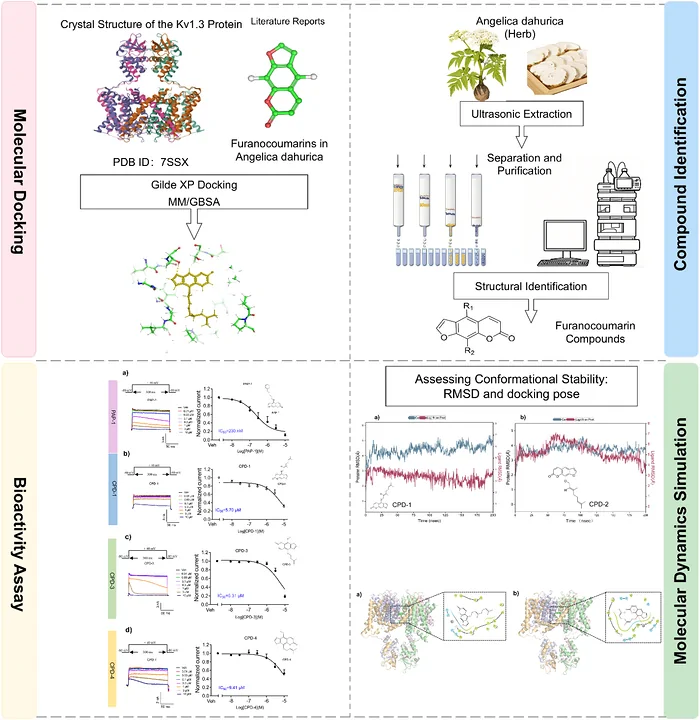

**Abbreviations**
CCK‐8: Cell Counting Kit‐8
CPD: compound
DDA: data‐dependent acquisition
DMSO: dimethyl sulfoxide
eGFP: Enhanced Green Fluorescent Protein
ESI: electrospray ionization
HEK‐293: Human Embryonic Kidney 293 cells
HPLC: high‐performance liquid chromatography
HRESI‐MS: high‐resolution electrospray ionization mass spectrometry
IC_50_
: half‐maximal inhibitory concentration
Kv1.3: Voltage‐Gated Potassium Channel 1.3
LB: Luria‐Bertani
LC‐MS: liquid chromatography–mass spectrometry
MD: molecular dynamics
MM/GBSA: molecular mechanics/generalized born surface area
MPLC: medium‐pressure preparative liquid chromatography
NMR: nuclear magnetic resonance
OPM: Orientations of Proteins in Membranes
PAP‐1: 5‐(4‐Phenoxybutoxy)psoralen
PBS: phosphate‐buffered saline
PDB: Protein Data Bank
PDD: cisplatin
PDL: poly‐D‐lysine
POPC: 1‐Palmitoyl‐2‐oleoyl‐sn‐glycero‐3‐phosphocholine
RMSD: root‐mean‐square deviation
RMSF: root‐mean‐square fluctuation
RRID: Research Resource Identifier
SAR: structure–activity relationship
SD: standard deviation
SEM: standard error of the mean
TIP3P: Transferable Intermolecular Potential with 3 Points
TLC: thin‐layer chromatography
UV: ultraviolet
XP: Extra Precision

## 1 Introduction

Since ancient times, medicinal plants have been used to manage and treat a wide range of diseases and conditions. Nature has therefore long been regarded as an important source of therapeutic agents [1]. Although synthetic drugs currently dominate clinical practice, research on medicinal herbs and their constituents remains highly relevant [2]. Plants continue to provide diverse pharmacologically active molecules that can serve as starting points for drug discovery, either through direct use or through structural modification and optimization [3].

One such plant is *Angelica dahurica* (Bai Zhi), a member of the Apiaceae family that holds an important place in traditional Chinese medicine [4]. First recorded in the Shennong Bencao Jing and classified as a middle‐grade herb [5], it has been used for centuries to treat headaches, rhinitis, toothache, leukorrhea, skin disorders, and various inflammatory conditions associated with pain [6]. Phytochemical studies have shown that the chemical profile of *A. dahurica* is complex, encompassing coumarins, essential oils, polysaccharides, alkaloids, and glycosides. Among these, furanocoumarins are recognized as the principal bioactive constituents. Modern pharmacological investigations have confirmed that both the crude extracts and isolated furanocoumarins of *A. dahurica* exhibit anti‐inflammatory, analgesic, antibacterial, antioxidant, and antitumor activities [7]. Despite this body of evidence, the molecular targets underlying many of these effects, particularly those related to immune and inflammatory regulation, remain incompletely defined.

A promising candidate target in this context is the voltage‐gated potassium channel Kv1.3. Highly expressed in effector memory T cells, Kv1.3 plays a pivotal role in T‐cell activation, immune responses, metabolic regulation, and neuroinflammation. It has consequently evolved from a subject of basic electrophysiological research into a validated therapeutic target for multiple disease categories, including autoimmune disorders, metabolic syndrome, neurodegenerative diseases, and cardiovascular complications [8, 9]. In 2005, Schmitz et al. reported PAP‐1, a synthetic 5‐phenoxyalkoxypsoralen derivative designed as a potent and selective Kv1.3 blocker [10]. Subsequent structure–activity relationship (SAR) studies demonstrated that the length of the alkoxy side chain, its hydrophobicity, and the substitution pattern on the terminal aromatic ring are critical determinants of both inhibitory potency and channel selectivity [11]. Although PAP‐1 and related synthetic compounds have provided valuable pharmacological tools, their clinical translation has been hindered by limitations in selectivity, metabolic stability, and practical routes of administration. These challenges have stimulated growing interest in the identification of novel Kv1.3 blockers from natural sources, particularly those sharing the furanocoumarin scaffold.

Natural‐product libraries, however, are often large and chemically diverse, making exhaustive experimental testing inefficient. Computational methods offer a practical solution. Molecular docking can rapidly evaluate the potential binding of hundreds of compounds to a target protein and predict preferred binding modes [12]. Subsequent end‐point energy calculations, such as MM/GBSA, and molecular‐dynamics simulations can further refine the ranking of candidates and provide atomic‐level insight into complex stability and key interactions [13]. When applied thoughtfully, these approaches streamline the discovery process, reduce experimental costs, and help prioritize the most promising molecules for laboratory validation.

Accordingly, this study adopted a computationally guided experimental strategy to identify natural Kv1.3 inhibitors from *A. dahurica*. (a) A library of furanocoumarin compounds from *A. dahurica* was constructed through a systematic literature survey, and virtual screening was performed using molecular docking and MM/GBSA calculations to evaluate their predicted interactions with the Kv1.3 channel. (b) Based on the virtual screening results, major furanocoumarins were isolated and purified from *A. dahurica* using MPLC and preparative HPLC, and subsequently analyzed and identified by LC–MS. Commercially available isomeric counterparts were included to complete the representative isomer pairs, and these isomers were then evaluated by whole‐cell patch‐clamp electrophysiology to verify their inhibitory activity against Kv1.3 and to determine their IC_50_ values; (c) Molecular dynamics simulations were performed to analyze the dynamic stability and key interactions of the ligand‐protein complex, elucidating at the atomic level the mechanism by which side‐chain substitution positions influence Kv1.3 inhibitory activity. The overall workflow of the study is summarized in (Figure 1).

**FIGURE 1:**
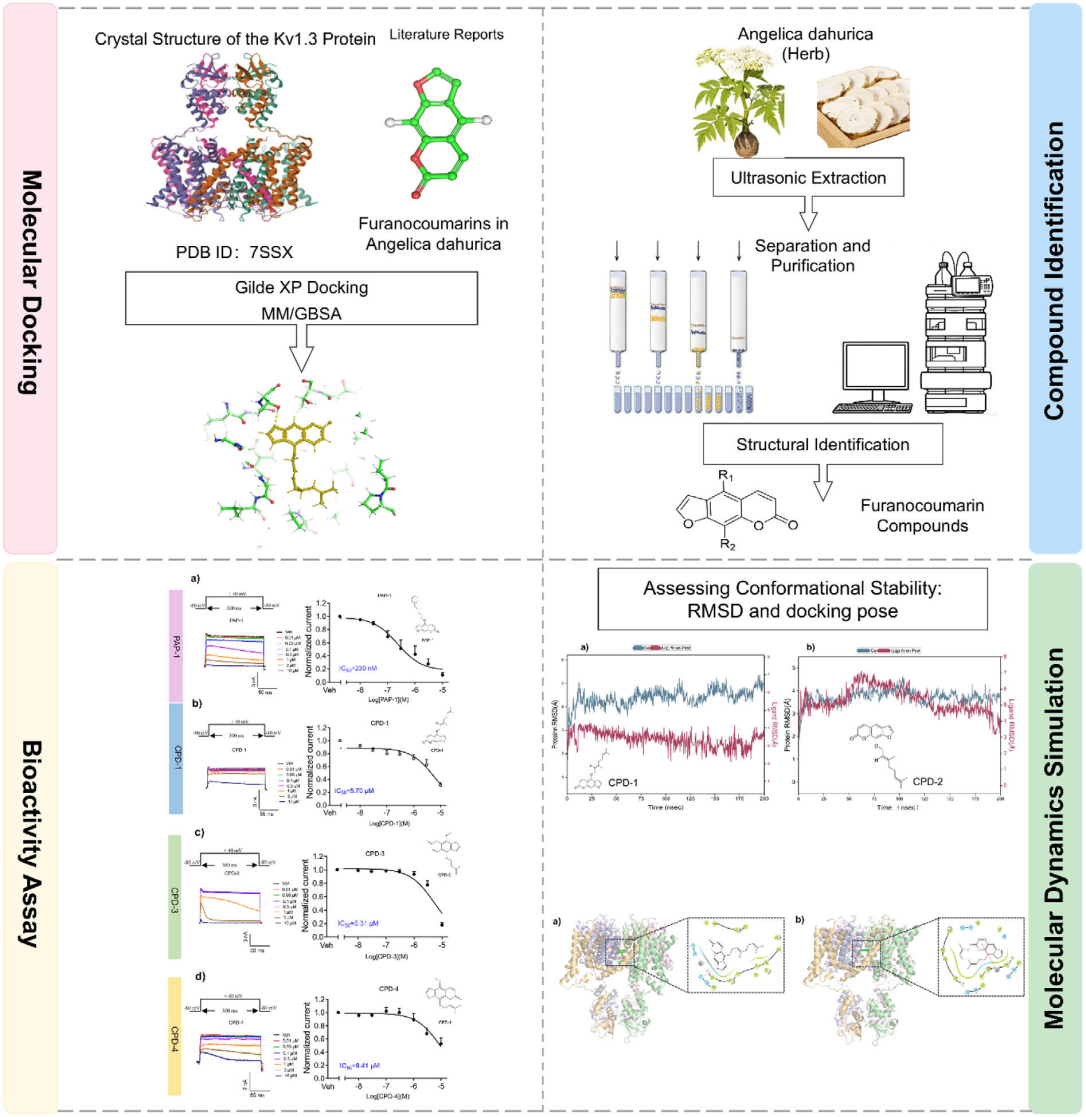
Schematic Diagram of Molecular Docking of Furanocoumarins in A. dahurica, Isolation and Extraction of A. dahurica Compounds, IC50 Determination, and RMSD and a 2D schematic diagram of the interaction between the compound and the protein.

## 2 Materials and Methods

### 2.1 Molecular Docking and MM/GBSA Calculations

The cryo‐EM structure of human Kv1.3 (PDB ID: 7SSX; resolution, 2.89 Å) was retrieved from the Protein Data Bank [14]. Molecular docking of 43 compounds derived from *A. dahurica* was performed using the Schrödinger Suite (version 2018). The structures of the compounds were drawn using ChemDraw, and the standard PAP‐1 structure was downloaded from PubChem in SDF format [15]. The Kv1.3 structure was prepared using the Protein Preparation Wizard, including assignment of bond orders, addition of hydrogen atoms, optimization of protonation states and the hydrogen‐bonding network, and restrained energy minimization using the OPLS3e force field; Epik was used to generate the predominant protonation and tautomeric states at pH 7.0 ± 2.0. All 43 ligands were prepared using the LigPrep module. The receptor grid, which defined the binding pocket, was generated using the Receptor Grid Generation module and centered at x = 136.32, y = 136.31, and z = 126.14 Å, with a box size of 20 × 20 × 20 Å [16]. The prepared ligand library and the aforementioned grid file were imported into the Glide module, and semi‐flexible docking was performed using the high‐precision XP (Extra Precision) mode [17]. The program output docking scores for each ligand for further analysis [18]. The molecular mechanics/generalized Born surface area (MM/GBSA) method was used to estimate relative ligand–protein interaction energies for comparative ranking [18, 19]. This calculation was performed in the Prime module of the Schrödinger software platform.

Conformations with favorable XP docking scores were selected, and MM/GBSA energy estimates were calculated using the Prime module for relative comparison [20]. In this approach, the energies of the ligand, receptor, and ligand–receptor complex were evaluated separately in an implicit solvent environment. The MM/GBSA energy estimate (ΔG_bind) was obtained from the difference between the energy of the ligand–receptor complex and the combined energies of the isolated receptor and ligand. A schematic diagram of the docked PAP‐1–Kv1.3 complex is shown in Figure 2.

**FIGURE 2:**
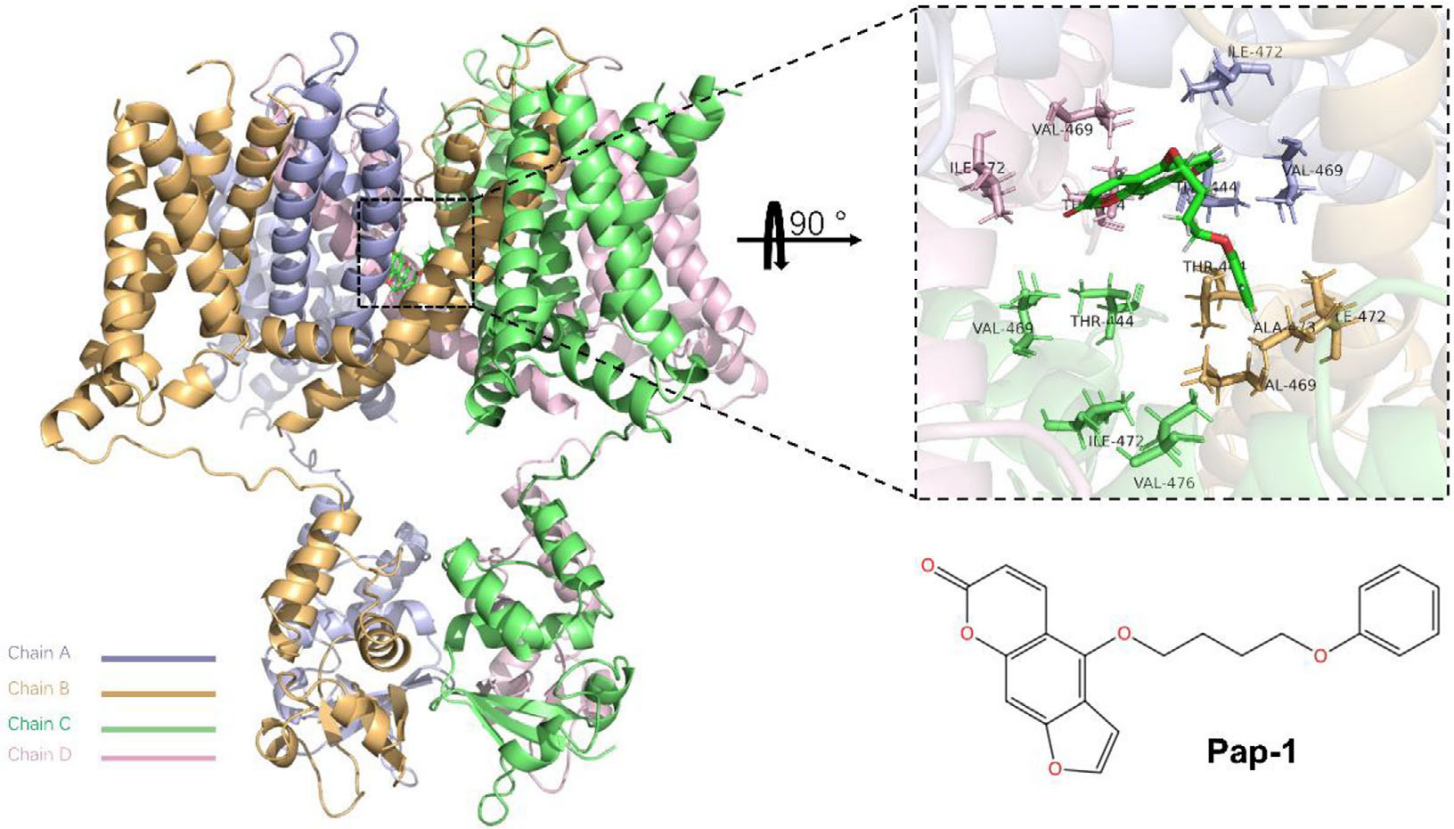
Schematic diagram of the docking conformation of the PAP‐1 and Kv1.3 proteins.

### 2.2 Chemicals

n‐Hexane, dichloromethane, ethyl acetate, methanol (Gaojing Chemical, AR Grade), methanol (Thermo Fisher, LC‐MS Grade), formic acid (Thermo Fisher, LC‐MS Grade), acetonitrile (SPECTRUME, HPLC Grade), water (Merck, LC‐MS Grade).

### 2.3 Plant Materials

Commercial dried root slices of *A. dahurica* were purchased via the Taobao online platform from Sichuan Hongya County Weikang Pharmaceutical Chain Co., Ltd. (Sichuan, China). The product was supplied under production license No. Chuan 20160063 and batch No. 250801.

### 2.4 Ultrasonic Extraction

The *A. dahurica* decoction pieces were ground and passed through a 60‐mesh sieve [21, 22]. A 100 g portion of the resulting powder was transferred to a beaker and extracted by ultrasound‐assisted extraction using a solid‐to‐liquid ratio of 1:12 (g/mL) at 30°C for 20 min per extraction [23, 24]. The extract was filtered through a Büchner funnel, and the residue was re‐extracted twice under the same conditions, resulting in a total of three extraction cycles [25]. The combined filtrates were concentrated under reduced pressure using a rotary evaporator, with the water bath temperature maintained below 40°C to prevent thermal degradation of heat‐sensitive compounds. Ultrasonic extraction was continued until the extract became colorless and TLC confirmed that the major constituents had been almost completely extracted [26]. Finally, the crude extract was obtained and weighed [27].

### 2.5 Liquid‐Liquid Extraction

The crude extract was suspended in 200 mL of distilled water, and approximately 5 mL of methanol was added dropwise to facilitate dissolution. The suspension was transferred to a separatory funnel and sequentially partitioned with equal volumes (200 mL) of n‐hexane, dichloromethane, and ethyl acetate [28]. Each solvent extraction was performed three times, and the corresponding organic phases were combined [29]. The n‐hexane, dichloromethane, and ethyl acetate fractions were concentrated to dryness under reduced pressure at a water‐bath temperature below 40°C. The resulting fractions were weighed, and their yields were recorded [30]. The remaining aqueous phase was freeze‐dried to obtain the water‐soluble fraction.

### 2.6 Thin‐Layer Chromatography (TLC)

A GF254 silica gel plate was prepared by drawing a starting line 1 cm from the lower edge and a solvent front line 0.5–1 cm from the upper edge. Sample solutions prepared in a volatile solvent were applied as small spots onto the starting line using a capillary tube. After the spots had dried, additional aliquots of the sample were applied as necessary to achieve an appropriate loading concentration. The spacing between adjacent spots was maintained at no less than 0.5 cm to prevent overlap during development. The developing solvent was n‐hexane/ethyl acetate (4:1, v/v). Approximately 10 mL of the developing solvent was added to the developing chamber, ensuring that the solvent level remained below the starting line. The chamber was covered and allowed to equilibrate for 10–15 min before the TLC plate was placed inside for development. Once the solvent front reached the marked front line, the plate was removed from the chamber and air‐dried. The separated spots were visualized under UV light at 254 and 365 nm, marked, and the corresponding retention factor (Rf) values were calculated.

### 2.7 Medium‐Pressure Preparative Liquid Chromatography

Separation was performed using a medium‐pressure preparative liquid chromatography system [31]. A silica gel column (40 g, 40–63 µm) was used as the stationary phase, and the mobile phase consisted of n‐hexane–ethyl acetate [32]. The gradient elution program was as follows: the volume fraction of ethyl acetate was increased from 0% to 100% over 0–35 min at a flow rate of 20 mL/min, with dual‐wavelength detection (254 and 310 nm) [33]. Samples were loaded using the dry method: the sample was dissolved in a small amount of volatile organic solvent and ground and mixed with silica gel (40–63 µm) in a mass ratio of approximately 1:1.5 to 1:2 [34]. After the solvent was evaporated under fume hood ventilation, the powder was evenly packed into the column head and covered with cotton wool [35]. Fractions were collected in full flow, 20 mL per tube. The corresponding fractions were combined based on the start and end retention times of the chromatographic peaks [5]. Each combined fraction was concentrated under reduced pressure to dryness at a temperature below 40°C [36]. Finally, the resulting fractions were analyzed by thin‐layer chromatography to guide subsequent purification [37].

### 2.8 LC–MS Instrumentation and Chromatographic Conditions

Chromatographic analysis was performed using a Hypersil Gold C18 column at a column temperature of 40°C and a flow rate of 0.2 mL/min. The mobile phase consisted of a 0.1% aqueous formic acid solution (A) and methanol (B), with gradient elution performed according to the following program: 0–1.5 min, 2% B; 1.5–3 min, phase B increased from 2% to 85%; 3–10 min, phase B increased from 85% to 100% and was held at that level; 10–10.1 min: Phase B was reduced to 2%; and from 10.1 to 12 min, 2% B was maintained to equilibrate the column. The total run time was 12 min [38]. m/z 100–1500; ESI: 3.5 kV spray voltage, 35 psi sheath gas, 10 L/min carrier gas; capillary 320°C; S‐lens RF 60; carrier gas heater 350°C; polarity: Pos, Neg; MS/MS in DDA mode.

### 2.9 Electrophysiological Assay of Kv1.3 Inhibition and IC50 Determination

The Kv1.3 plasmid (1 µL, 2 ng/µL) was transformed into TransBlue‐10 competent cells (50–100 µL) by incubation on ice for 30 min, followed by heat shock at 42°C for 45 s (or 37°C for 90 s) and a subsequent 2 min incubation on ice. Subsequently, 400 µL of antibiotic‐free LB medium was added, and the cells were incubated at 37°C with shaking at 200 rpm for 1 h to allow recovery. The transformed cells were then spread onto LB agar plates containing 20 µg/mL ampicillin, air‐dried in a laminar flow hood for 30 min, and incubated overnight at 37°C in an inverted position. Single colonies were selected on the following day and inoculated into 20 mL of LB medium supplemented with ampicillin. After incubation at 37°C with shaking at 200 rpm for approximately 16 h, plasmids were extracted using the GoldHi EndoFree Plasmid Midi Kit [39]. Frozen HEK‐293 cells were rapidly thawed in a 37°C water bath and transferred to a 15‐mL centrifuge tube containing 5–10 mL of pre‐warmed complete medium. Following centrifugation at 800 rpm for 3–5 min, the supernatant was discarded, and the cells were resuspended in complete medium and cultured at 37°C in a humidified incubator containing 21% O_2_ and 5% CO_2_. The culture medium was replaced 24 h after cell attachment. When the cells reached 80%–90% confluence, they were washed three times with PBS and detached using pre‐warmed 0.05% trypsin for approximately 60 s at room temperature. The detached cells were resuspended in complete medium and passaged at a ratio of 1:3–1:5 every 2–3 days. Cells after three passages were used for transfection. Prior to transfection, the cells were harvested, counted, and seeded into 35‐mm culture dishes pre‐coated with poly‐D‐lysine (PDL, 10 µg/mL) at a density of 1 × 10^6^ cells/mL [40]. After overnight incubation to approximately 70% confluence, the culture medium was replaced with 1 mL of serum‐free Opti‐MEM medium, and the cells were serum‐starved for 1 h. Transfection Solution A consisted of 250 µL Opti‐MEM containing 1 µg eGFP plasmid and 3–6 µg Kv1.3 plasmid, whereas Solution B consisted of 250 µL Opti‐MEM containing 4 µL Lipofectamine 2000. After standing separately for 5 min, the two solutions were mixed and incubated at room temperature for 30 min before being added dropwise to the culture dishes. The cells were incubated at 37°C in 5% CO_2_ for 6–10 h, then detached and replated into several 35‐mm culture dishes at a suitable density to allow individual cell attachment. After an additional 12 h of culture, the cells were used for electrophysiological recordings.

Electrophysiological experiments were recorded at room temperature (approximately 25°C) using a HEKA EPC‐10 amplifier. Prior to the experiment, adherent cells were gently washed three times with extracellular solution preheated to 37°C; the final bath volume was 1.5 mL. Glass electrodes were prepared using a puller, filled with internal solution to a point 1–1.5 cm from the tip, and mounted in an electrode holder after air bubbles were removed. The micro‐manipulation control system was used to guide the electrode toward the cell at high speed (settings 0–3) before immersion, medium speed (settings 4–6) during immersion, and low speed (settings 7–9) near the cell. The resistance upon immersion was 3–5 MΩ. After the electrode made contact with the cell, it was pressed downward until the resistance increased by 0.2 MΩ; a slow, sustained negative voltage was then applied until the sealing resistance reached approximately 1 GΩ. This was followed by a brief, strong negative voltage to rupture the membrane. Fast and slow capacitances, series resistance, and leakage current were compensated for, and the gain was adjusted. After stabilizing for 3–5 min, drug administration and recording began.

Two voltage‐clamp protocols were employed. For the step‐voltage protocol, currents were sampled at 5 kHz while cells were held at −80 mV for 20 ms and depolarized from −100 to +80 mV in 10 mV increments for 50 ms. The maximum outward current at each voltage step was recorded. For the single‐voltage protocol, currents were sampled at 5 kHz while cells were held at −80 mV for 100 ms, depolarized to +20 mV for 300 ms, and then repolarized to −80 mV. The maximum outward current at +20 mV was used for subsequent analysis.

#### 2.9.1 Cell Culture

HEK‐293 cells (RRID:CVCL_0045) were purchased from Shanghai Fuheng Biotechnology Co., Ltd. (Shanghai, China), whereas RAW 264.7 mouse monocyte/macrophage cells (RRID:CVCL_0493) and L929 mouse fibroblast cells (RRID:CVCL_0462) were purchased from Zhejiang Meisen Cell Technology Co., Ltd. (Zhejiang, China). All three cell lines were cultured in Dulbecco's Modified Eagle Medium (DMEM) supplemented with 10% fetal bovine serum (FBS) and 1% penicillin–streptomycin and maintained at 37°C in a humidified incubator containing 5% CO_2_.

**Cell Thawing**: Frozen cells were removed from liquid nitrogen and rapidly thawed in a 37°C water bath with gentle agitation for approximately 1 min. The cell suspension was transferred to a centrifuge tube containing 9 mL of complete culture medium and centrifuged at 1000 rpm for 3 min. After removal of the supernatant, the cell pellet was resuspended in 1 mL of fresh complete medium and seeded into a 5‐cm culture dish. The dish was gently swirled to ensure even cell distribution and incubated at 37°C in a humidified incubator containing 5% CO_2_. The culture medium was replaced after 24–48 h according to cell density.

**Cell Passaging**: Cell morphology and confluence were routinely monitored under an inverted microscope, and cells were passaged when confluence reached approximately 90% [41]. The culture medium was removed, and the cells were washed twice with pre‐warmed PBS. Approximately 2 mL of trypsin solution was added, and the cells were digested for 1–2 min at 37°C or room temperature until they became rounded and detached. Digestion was terminated by adding an equal volume of serum‐containing medium. Because RAW 264.7 macrophages are semi‐adherent and highly sensitive to trypsin, enzymatic digestion was omitted during passaging. Instead, the cells were washed with PBS, detached using a cell scraper or gentle pipetting, centrifuged, resuspended, and subcultured at a ratio of 1:3 to 1:4.

**Cell Cryopreservation**: Cells in the logarithmic growth phase with 80%–90% confluence were collected for cryopreservation. Following PBS washing, the cells were digested with approximately 2 mL of trypsin for 1–2 min at 37°C and neutralized with an equal volume of serum‐containing medium. The detached cells were collected by centrifugation at 1000 rpm for 3 min, resuspended in cryopreservation medium containing 10% DMSO, and adjusted to a final density of 5 × 10^6^–1 × 10^7^ cells/mL [41]. Aliquots (1–1.5 mL) were transferred into cryovials, labeled appropriately, stored overnight at −80°C in a programmable freezer, and subsequently transferred to liquid nitrogen for long‐term storage.

#### 2.9.2 CCK‐8 Cell Viability Assay

Cells at 85%–95% confluence were harvested and prepared as a single‐cell suspension by centrifugation. After cell counting, the suspension was adjusted to a density of 8,000 cells per 100 µL, and 100 µL was seeded into each well of a 96‐well plate. The cells were incubated at 37°C in a humidified incubator containing 5% CO_2_ for 16–20 h to allow cell attachment [41]. The culture medium was then replaced with fresh medium containing 10 µM of the test compound, and the cells were further incubated for 24 h. After treatment, the medium was removed, and 100 µL of fresh culture medium containing 10% CCK‐8 reagent was added to each well. Following incubation for 1–2 h, absorbance was measured at 450 nm using a microplate reader. Wells containing culture medium alone served as the negative control, while cisplatin‐treated wells served as the positive control. Each experiment was performed with at least three independent replicates, and the data are presented as the mean ± SD. Statistical analysis and graph preparation were performed using Origin 2018.

### 2.10 Molecular Dynamics Studies

All‐atom molecular dynamics (MD) simulations were performed using the Desmond module in Schrödinger Suite 2022‐4 under a Linux environment. The docked Kv1.3–ligand complexes were used as initial structures, and the membrane orientation of Kv1.3 was determined using the PPM 3.0 tool implemented in the Orientations of Proteins in Membranes (OPM) server with default settings. The complexes were prepared with the Protein Preparation Wizard, including optimization of protonation states at pH 7.0 ± 2.0, hydrogen‐bonding networks, and restrained minimization using the OPLS4 force field. Simulation systems were constructed using the System Builder module with a POPC lipid bilayer and TIP3P water model, maintaining a solvent buffer of at least 10 Å from the protein surface. Na^+^ and Cl^−^ ions were added to neutralize the system and achieve an ionic concentration of approximately 0.15 M. MD simulations were performed under the NPT ensemble at 300 K and 1 atm using a Nosé–Hoover chain thermostat and Martyna–Tobias–Klein barostat, respectively. Each system underwent the standard restrained relaxation and equilibration protocol implemented in Desmond, followed by a 200 ns production simulation using an integration time step of 2 fs.

## 3 Results

### 3.1 Molecular Docking and MM/GBSA Calculations

In this study, PAP‐1 was used as the positive control compound, with a docking score of −6.510 and an MM/GBSA energy estimate of −40.94. Figure 3 shows the two‐dimensional structures of 43 furanocoumarin compounds derived from *A. dahurica*; these structures were drawn using ChemDraw (version: ChemDraw 23.1.1). Table 1 lists the docking scores and MM/GBSA scores for these 43 compounds.

FIGURE 3Two‐dimensional structures of the 43 furanocoumarins derived from *A. dahurica*, and of PAP‐1.

**Figure:**
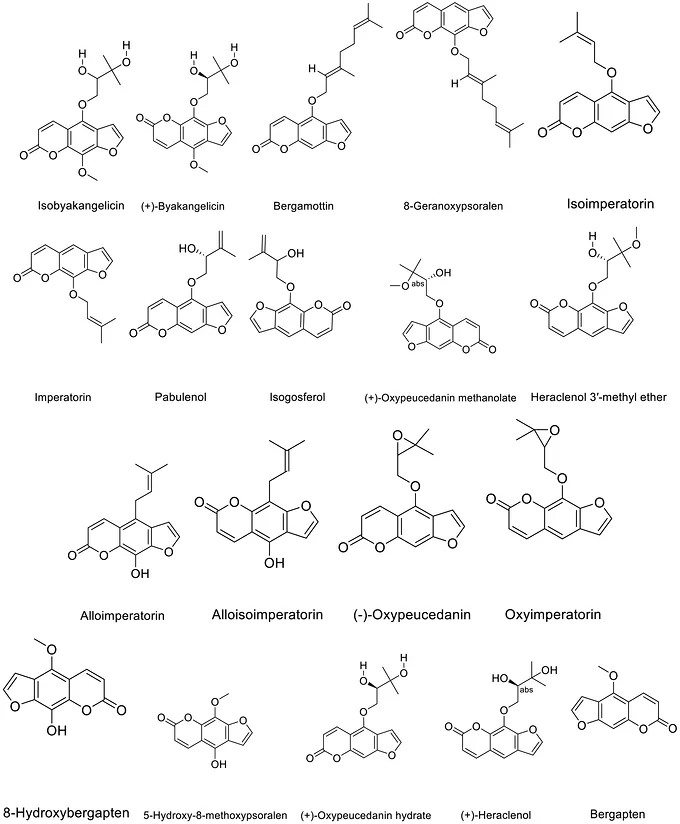

**Figure:**
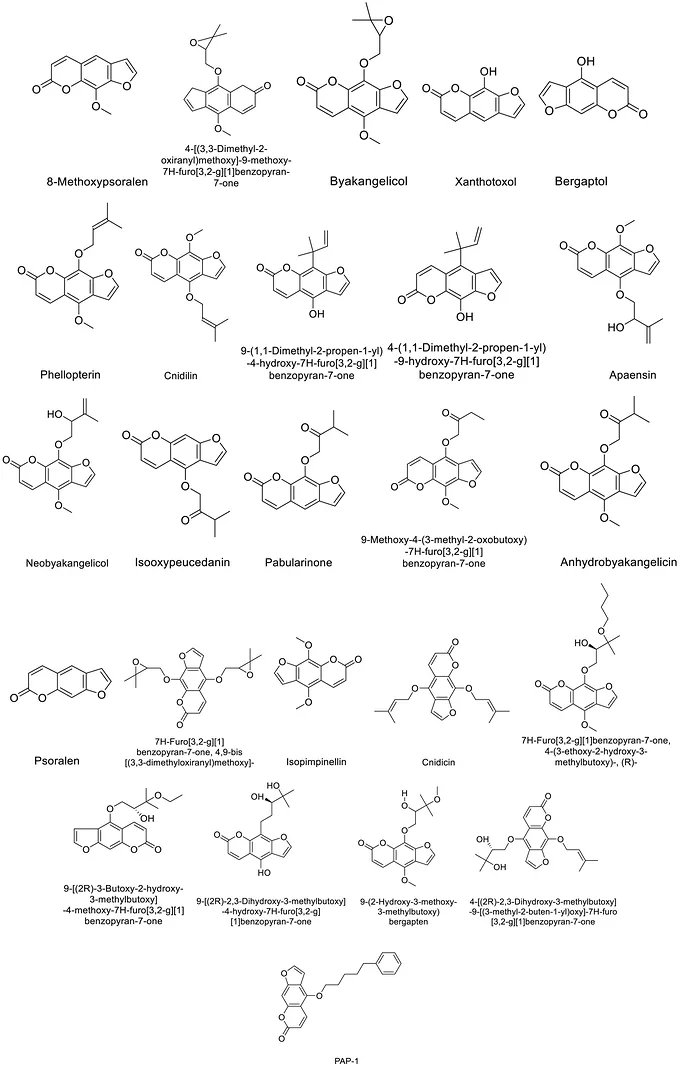

**TABLE 1:** Docking scores (XP) and MM/GBSA dG Bind scores for 43 compounds.

Compound name	CAS	MW (g/mol)	Docking score (kcal/mol)	MM/GBSA (kcal/mol)
Isobyakangelicin	50791‐60‐1	334.32	−6.55	−34.83
(+)‐Byakangelicin	482‐25‐7	334.32	−5.28	−11.73
Bergamottin	7380‐40‐7	338.4	−6.51	−34.30
8‐geranyloxypsoralen	71612‐25‐4	338.4	−6.32	−16.53
Isoimperatorin	482‐45‐1	270.28	−6.41	−29.85
Imperatorin	482‐44‐0	270.28	−5.77	−9.83
Pabulenol	33889‐70‐2	286.28	−6.24	−39.18
Isogosferol	53319‐52‐1	286.28	−5.99	−28.51
(+)‐Oxypeucedanin methanolate	52939‐12‐5	318.32	−6.20	−31.68
Heraclenol 3′‐methyl ether	31456‐78‐7	318.32	−5.83	−26.37
Alloimperatorin	642‐05‐7	270.28	−5.80	−32.26
Alloisoimperatorin	35214‐83‐6	270.28	−5.67	−33.71
(‐)‐Oxypeucedanin	26091‐73‐6	286.28	−6.13	−32.64
Oxyimperatorin	35740‐18‐2	286.28	−5.76	−15.00
8‐Hydroxybergapten	1603‐47‐0	232.19	−6.11	−26.38
5‐Hydroxy‐8‐methoxypsoralen	7471‐73‐0	232.19	−5.71	−26.43
(+)‐Oxypeucedanin hydrate	2643‐85‐8	304.3	−6.07	−36.11
(+)‐Heraclenol	31575‐93‐6	304.3	−5.67	−22.98
Bergapten	484‐20‐8	216.19	−6.03	−24.75
8‐Methoxypsoralen	298‐81‐7	216.19	−5.35	−9.34
4‐[(3,3‐Dimethyl‐2‐oxiranyl)methoxy]‐9‐methoxy‐7H‐furo[3,2‐g][1]benzopyran‐7‐one	50275‐25‐7	316.31	−5.99	−22.42
Byakangelicol	26091‐79‐2	316.31	−5.97	−17.41
Xanthotoxol	2009‐24‐7	202.16	−5.99	−14.98
Bergaptol	486‐60‐2	202.16	−5.17	−17.53
Phellopterin	2543‐94‐4	300.31	−5.44	−14.07
Cnidilin	14348‐22‐2	300.31	−5.71	−19.80
9‐(1,1‐Dimethyl‐2‐propen‐1‐yl)‐4‐hydroxy‐7H‐furo[3,2‐g][1]benzopyran‐7‐one	60924‐68‐7	270.28	−5.68	−14.77
4‐(1,1‐Dimethyl‐2‐propen‐1‐yl)‐9‐hydroxy‐7H‐furo[3,2‐g][1]benzopyran‐7‐one	89329‐26‐0	270.28	−5.57	−11.59
Apaensin	80508‐43‐6	316.31	−5.97	−24.80
Neobyakangelicol	35214‐82‐5	316.31	−5.32	−7.57
Isooxypeucedanin	5058‐15‐1	286.28	−5.41	−11.22
Pabularinone	4889‐31‐0	286.28	−5.61	−10.64
9‐Methoxy‐4‐(3‐methyl‐2‐oxobutoxy)‐7H‐furo[3,2‐g][1]benzopyran‐7‐one	1253373‐70‐4	316.31	−5.45	−18.48
Anhydrobyakangelicin	35214‐81‐4	316.31	−5.57	−16.51
Psoralen	66‐97‐7	186.16	−5.90	−18.72
7H‐Furo[3,2‐g][1]benzopyran‐7‐one, 4,9‐bis[(3,3‐dimethyloxiranyl)methoxy]‐	77063‐74‐2	386.4	−6.13	−19.54
Isopimpinellin	482‐27‐9	246.22	−5.66	−10.29
Cnidicin	14348‐21‐1	354.4	−5.59	−11.48
7H‐Furo[3,2‐g][1]benzopyran‐7‐one, 4‐(3‐ethoxy‐2‐hydroxy‐3‐methylbutoxy)‐, (R)‐	90686‐46‐7	332.35	−5.65	−34.70
9‐[(2R)‐3‐Butoxy‐2‐hydroxy‐3‐methylbutoxy]‐4‐methoxy‐7H‐furo[3,2‐g][1]benzopyran‐7‐one	708266‐25‐5	390.43	−5.33	−15.07
9‐[(2R)‐2,3‐Dihydroxy‐3‐methylbutoxy]‐4‐hydroxy‐7H‐furo[3,2‐g][1]benzopyran‐7‐one	663602‐89‐9	320.29	−4.78	−13.20
9‐(2‐Hydroxy‐3‐methoxy‐3‐methylbutoxy)bergapten	79638‐04‐3	348.35	−5.83	−20.90
4‐[(2R)‐2,3‐Dihydroxy‐3‐methylbutoxy]‐9‐[(3‐methyl‐2‐buten‐1‐yl)oxy]‐7H‐furo[3,2‐g][1]benzopyran‐7‐one	708266‐24‐4	388.41	−5.82	−41.96

### 3.2 LC‐MS Analysis and Preliminary Identification of Furanocoumarin Compounds

After crude extraction and further extraction of *A. dahurica*, four polar fractions were obtained: n‐hexane, dichloromethane, ethyl acetate, and water. TLC analysis showed that the aqueous phase had the highest polarity, with spots firmly adsorbed on the silica gel and remaining at the origin without separation; the n‐hexane phase had the lowest polarity, with spots migrating significantly with the developing solvent; the dichloromethane phase had moderate polarity, with most spots showing good separation; and the ethyl acetate phase had a polarity intermediate between those of the aqueous phase and dichloromethane.

LC‐MS analysis was performed on the crude extract of *A. dahurica*, the n‐hexane extract of the crude extract, and the dichloromethane extract of the crude extract. To cover different polarities, LC‐MS detection was conducted in both positive and negative ion modes. In positive ion mode, the following compounds were detected: Heraclenin (m/z 269.081, [M+H‐H_2_O]^+^), Byakangelicol (m/z 299.0915, [M+H‐H_2_O]^+^), Isopimpinellin (m/z 247.06, [M+H]^+^), (Rac)‐Byakangelicin (m/z 317.1018, [M+H‐H_2_O]^+^), Imperatorin (m/z 271.0965, [M+H]^+^), Phellopterin (m/z 301.107, [M+H]^+^), Bergapten (m/z 217.0494, [M+H]^+^), (‐)‐Oxypeucedanin hydrate (m/z 305.1017, [M+H]^+^), Oxypeucedanin (m/z 287.0912, [M+H]^+^), and Isoimperatorin (m/z 271.0964, [M+H]^+^); In negative ion mode, the following compounds were detected: Nodakenetin (m/z 245.0819, [M‐H]^−^), Smyrindioloside (m/z 423.1295, [M‐H]^−^), Bergaptol (m/z 201.0193, [M‐H]^−^), and Alloimperatorin (m/z 269.0817, [M‐H]^−^), as shown in (Figure S1a,b). Combining the results from both modes, a total of 14 major furanocoumarins were identified. The results are shown in (Table S1).

### 3.3 Separation and Purification of Components From n‐Hexane and Dichloromethane Extracts of *A. dahurica*

The n‐hexane extract of *A. dahurica* was separated by medium‐pressure liquid chromatography (MPLC) (Figure S2). Based on the chromatographic peak ranges, the consecutively collected eluates were combined into eight fractions, designated Z1–Z8. The obtained masses of fractions Z2–Z8 were 130, 160, 51, 18, 8.7, 9.8, and 10 mg, respectively.

The components obtained by MPLC were further purified using preparative HPLC (Figure S3, detection wavelength 310 nm, flow rate 8.0 mL/min): Z2 (130 mg) was eluted isocratically with 70% acetonitrile to yield Z‐S1 (28 mg, tR = 19.86 min); Z3 (160 mg) was eluted isocratically with 65% acetonitrile to yield Z‐06 (11.9 mg, tR = 12.02 min), Z‐07 (25.5 mg, tR = 19.02 min), and Z‐08 (28.4 mg, tR = 21.49 min); Z4 (51 mg) was eluted isocratically with 60% acetonitrile to yield Z‐09 (11 mg, tR = 19.87 min); Z5 (18 mg) yielded Z‐10 (9 mg, tR = 15.48 min; no separate preparation gradient was noted). Each compound was analyzed by HPLC (0–30 min, 30%–100% acetonitrile gradient, 2.0 mL/min, 310 nm); the chromatograms are shown in (Figure S4).

The components of the dichloromethane extract of *A. dahurica* were separated and purified using medium‐pressure preparative liquid chromatography; the chromatogram is shown in (Figure S5). Based on the merging of chromatographic peaks, five components (E1–E5) were identified. TLC analysis revealed that component E5 contained a relatively large number of compounds, with deep‐colored and large spots, and a distinct main component.

Using preparative HPLC (30% acetonitrile isocratic, 8.0 mL/min, 310 nm), E‐5‐1 (8.1 mg, tR = 34.92 min) and E‐5‐2 (4.2 mg, tR = 37.63 min) were purified from E5 (81.2 mg). Analysis of the HPLC chromatogram (0–30 min, 30%–100% acetonitrile gradient, 2.0 mL/min, 310 nm) are shown in (Figure S6).

Systematic identification of furanocoumarin compounds via ultraviolet‐visible (UV) spectroscopy, high‐resolution mass spectrometry (HRESI‐MS), and nuclear magnetic resonance (^1^H NMR, ^1^ ^3^C NMR) spectroscopy.

To support structural identification, this study provides the NMR spectra and data assignment for all compounds. The ^1^H NMR and ^1^ ^3^C NMR spectra of Z‐S1, Z‐06, Z‐07, Z‐08, Z‐09, Z‐10, E‐5‐1, and E‐5‐2 are shown in Supplementary (Figures S7–S14), respectively, and the data assignments are summarized in (Tables S2–S9).

### 3.4 Electrophysiological Experiments to Verify Inhibitory Activity and IC50 Determination

The objective of this study was to identify inhibitors of the Kv1.3 potassium channel from 43 furanocoumarin isomers derived from A. dahurica. Based on a comprehensive evaluation of docking scores, MM/GBSA estimates, and compound availability, four pairs (a total of eight compounds) were selected for experimental validation. These included CPD‐5 (Isoimperatorin, isolated as Z‐S1), CPD‐6 (Imperatorin, isolated as Z‐07), CPD‐4 (Phellopterin, isolated as Z‐08), and CPD‐7 ((−)‐oxypeucedanin, isolated as Z‐09). The purities of CPD‐4, CPD‐5, CPD‐6, and CPD‐7 were 92.15%, 96.33%, 94.31%, and 94.95%, respectively, as determined by analytical HPLC using peak‐area normalization. The remaining four compounds, CPD‐1 (Bergamottin), CPD‐2 (8‐geranyloxypsoralen), CPD‐3 (Cnidilin), and CPD‐8 (Oxyimperatorin), were purchased from Taoshu Biotechnology Co., Ltd., with reported purities of ≥98%. Using whole‐cell patch‐clamp recordings, the inhibition of Kv1.3 currents by the eight compounds was first evaluated at a screening concentration of 10 µM, with PAP‐1 used as the positive control. The results are summarized in Table 2 and Figure S15, and the corresponding chemical structures are shown in Figure 4. These single‐concentration measurements were used as an initial assessment of Kv1.3 inhibition and were not interpreted as direct measures of potency. Concentration–response experiments were subsequently performed to determine the IC_50_ values of PAP‐1 and CPD‐1–CPD‐8. PAP‐1 showed an IC_50_ value of 230 nM (Figure 5a), while CPD‐1 (Bergamottin), CPD‐3 (Cnidilin), and CPD‐4 (phellopterin) exhibited IC_50_ values of 5.70, 5.31, and 9.41 µM, respectively (Figure 5b–d). The remaining compounds yielded IC_50_ values of 22.42 µM for CPD‐2, 7.04 µM for CPD‐5, 4.27 µM for CPD‐6, 8.26 µM for CPD‐7, and 19.81 µM for CPD‐8. The concentration–response curves and IC_50_ values for all eight compounds (CPD‐1‐CPD‐8) are presented together (Figure S16a–h). Accordingly, comparisons of compound potency were based on the concentration–response‐derived IC_50_ values rather than on the inhibition measured at the single screening concentration of 10 µM.

**TABLE 2:** Inhibitory activity of compounds against Kv1.3 potassium channel at 10 µM.

Pairs	Compound	Inhibition Rate (%)	Concentration (µM)
Positive Control	PAP‐1	99.7 ± 9.97	10
Pair 1	CPD‐1	96.1 ± 5.63	10
	CPD‐2	19.7 ± 5.85	10
Pair 2	CPD‐3	78.7 ± 13.29	10
	CPD‐4	75.7 ± 4.73	10
Pair 3	CPD‐5	58.2 ± 5.98	10
	CPD‐6	41.2 ± 9.97	10
Pair 4	CPD‐7	52.3 ± 6.80	10
	CPD‐8	32.4 ± 18.79	10

**FIGURE 4:**
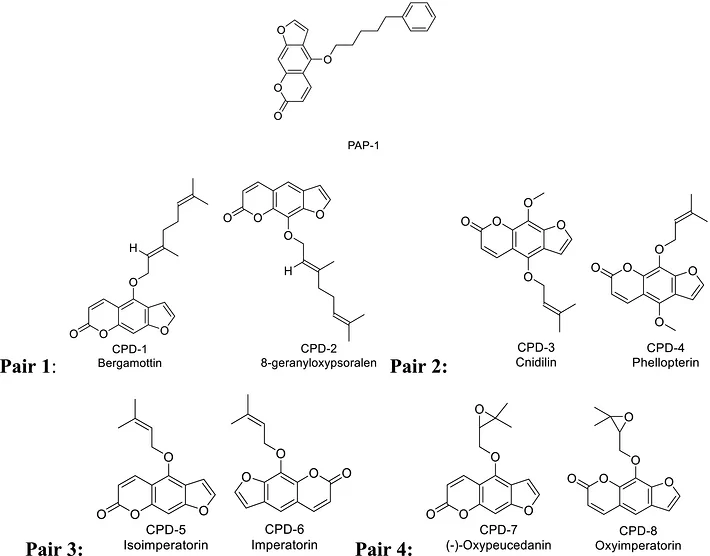
Schematic diagrams of the chemical structures of the positive control PAP‐1 and CPD‐1 to CPD‐8.

**FIGURE 5:**
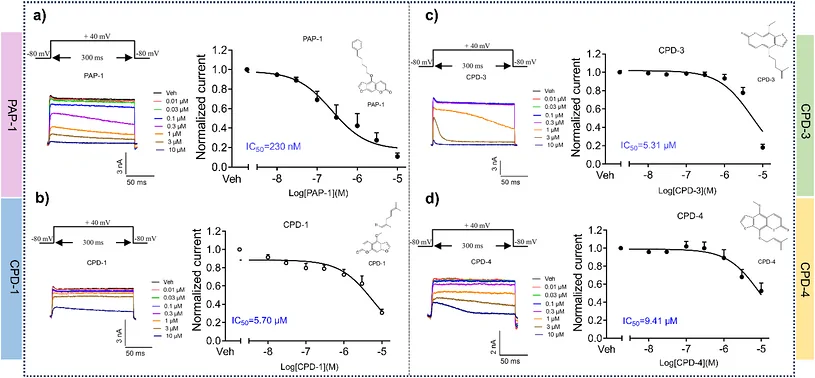
IC_50_ values for PAP‐1 and three candidate compounds determined by electrophysiological experiments. (a) PAP‐1, (a) IC50 of PAP‐1 against Kv1.3. (b) IC50 of CPD‐1 (Bergamottin) against Kv1.3. (c) IC50 of CPD‐3 (Cnidilin) against Kv1.3 (d) IC50 of CPD‐4 (Phellopterin) against Kv1.3.

### 3.5 Compound Toxicity Studies

The cytotoxicity of CPD‐1, CPD‐3, and CPD‐4 was assessed by CCK‐8 assay, with cisplatin (PDD) as the positive control. After 24 h of treatment at 10 µM, cell viability in L929 mouse fibroblasts exceeded 85% for all three compounds, whereas cisplatin (PDD) at the same concentration reduced viability to 41.06% (Figure S17a). Under the same 24 h treatment conditions, viability in RAW 264.7 mouse monocyte/macrophage cells exceeded 80% for all three compounds, compared with 29.01% for cisplatin (PDD) (Figure S17b). These results indicate that, at 10 µM, the three furanocoumarins did not exhibit significant cytotoxicity in either L929 fibroblasts or RAW 264.7 macrophages.

### 3.6 Statistical Analysis

Electrophysiological data were acquired using PatchMaster software and analyzed using Origin 8 and GraphPad Prism 6.0. Electrophysiological data are presented as the mean ± standard error of the mean (SEM), with error bars representing SEM. For the concentration–response experiments of PAP‐1 and CPD‐1–CPD‐8, five independently recorded cells were analyzed at each concentration (n = 5). Concentration–response curves were fitted by nonlinear regression, and IC_50_ values were calculated from the fitted curves. These concentration–response analyses were used for IC_50_ estimation rather than for inferential comparisons between treatment groups; therefore, *p* values and significance symbols are not applicable to these curves. Where comparisons among multiple groups were performed for the electrophysiological experiments, one‐way analysis of variance (ANOVA) was used, with p < 0.05 considered statistically significant.

For the CCK‐8 cell viability assays, data are presented as the mean ± standard deviation (SD) from three independent experiments (n = 3), with error bars representing SD. Statistical analysis and graph preparation were performed using Origin 2018. No inferential statistical comparisons were performed for the CCK‐8 assays; therefore, *p* values and significance symbols are not applicable.

### 3.7 Molecular Dynamics Study

To further investigate the binding stability of the four compounds, 200‐ns molecular dynamics simulations were performed. Root‐mean‐square deviation (RMSD), root‐mean‐square fluctuation (RMSF), and protein–ligand interaction analyses were conducted on the resulting trajectories. (Figure 6) clearly illustrates 2D schematic diagrams of the interactions between the compounds and the protein during the docking process for CPD‐1(Bergamottin) (Figure 6a), CPD‐2(8‐geranyloxypsoralen) (Figure 6b), CPD‐3(Cnidilin) (Figure 6c), and CPD‐4(Phellopterin) (Figure 6d). Figure S18 clearly illustrates the position of the compound within the protein during the Kv1.3 docking process.

**FIGURE 6:**
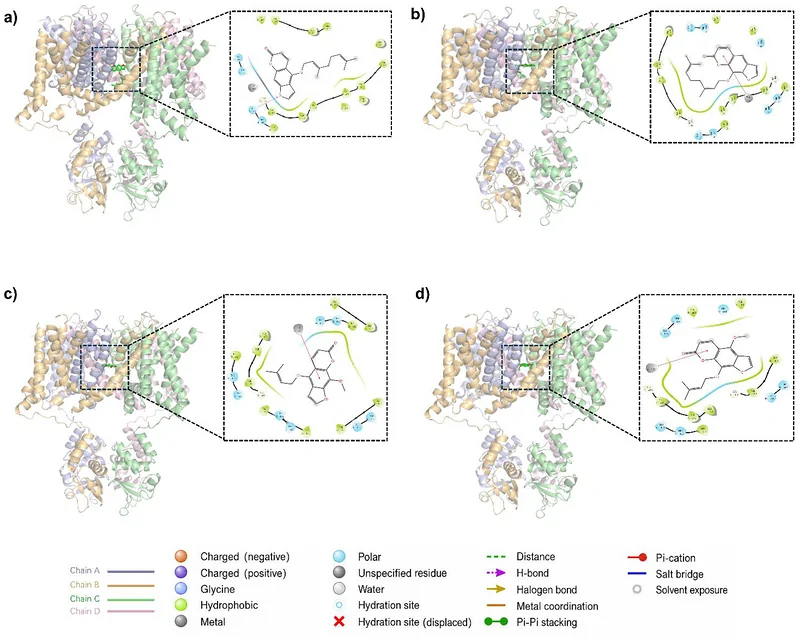
Two‐dimensional protein–ligand interaction diagrams of the selected compounds with Kv1.3. **(a)** CPD‐1 (Bergamottin)–Kv1.3 complex. **(b)** CPD‐2 (8‐geranyloxypsoralen)–Kv1.3 complex. **(c)** CPD‐3 (Cnidilin)–Kv1.3 complex. **(d)** CPD‐4 (Phellopterin)–Kv1.3 complex.

### 3.8 RMSD

During the 200 ns molecular dynamics simulations, the four Kv1.3–ligand complexes exhibited distinct RMSD profiles (Figure 7). For the CPD‐1 (bergamottin)–Kv1.3 complex, the protein Cα RMSD increased during the initial stage of the simulation and subsequently remained mainly within 3.5–5.0 Å (Figure 7a). The ligand RMSD also underwent an early conformational adjustment, after which it remained relatively stable without a sustained increase. For the CPD‐2 (8‐geranyloxypsoralen)–Kv1.3 complex, the ligand RMSD showed greater fluctuations and several plateau transitions, reaching approximately 5.0–7.0 Å before decreasing to around 4.8 Å during the later stage of the simulation (Figure 7b). These changes suggest greater conformational variability than that observed for CPD‐1. For the CPD‐3 (cnidilin)–Kv1.3 complex, the protein Cα RMSD remained mainly within 4.0–5.0 Å after the initial stage, whereas the ligand RMSD remained largely within 5.5–7.0 Å without a sustained upward trend (Figure 7c). This relatively stable RMSD profile was consistent with the experimentally observed Kv1.3 inhibition of CPD‐3 and its IC_50_ value of 5.31 µM. For the CPD‐4 (phellopterin)–Kv1.3 complex, the protein Cα RMSD remained approximately within 4.0–5.0 Å, whereas the ligand RMSD exhibited larger fluctuations and transitions between different RMSD plateaus (Figure 7d). Overall, the protein Cα RMSD profiles showed relative stabilization during the later stages of the 200 ns simulations, while the ligand RMSD profiles exhibited different degrees of conformational variability among the four Kv1.3–ligand complexes.

**FIGURE 7:**
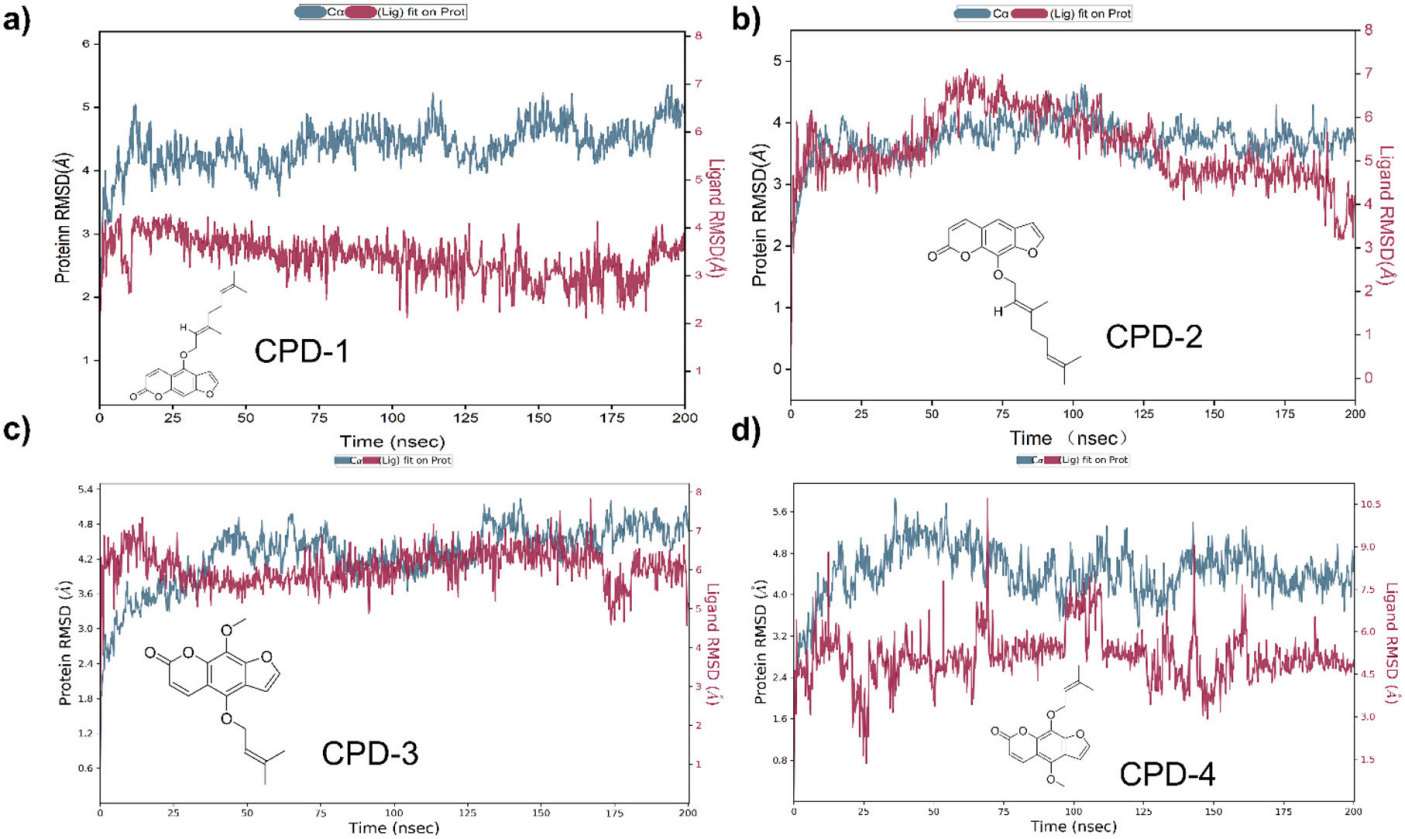
Root‐mean‐square deviation (RMSD) of the complexes calculated from 200 ns molecular dynamics simulations. The y‐axis represents the RMSD values. The x‐axis represents simulation time (in nanoseconds). (a) RMSD values for the CPD‐1 (Bergamottin)–Kv1.3 channel complex; (b) RMSD values for the CPD‐2 (8‐geranyloxypsoralen)–Kv1.3 channel complex; (c) RMSD values for the CPD‐3 (Cnidilin)–Kv1.3 channel complex; (d) RMSD values for the CPD‐4 (Phellopterin)–Kv1.3 channel complex.

### 3.9 RMSF

RMSF analysis revealed differences in local protein flexibility among the four Kv1.3–ligand complexes (Figure 8). In the CPD‐1 (Bergamottin) system, most residues exhibited RMSF values of approximately 0.8–2.0 Å, and the ligand‐binding residues indicated by the green vertical lines were predominantly located in regions of relatively low fluctuation (Figure 8a), suggesting a stable local environment around the binding region. In the CPD‐2 (8‐geranyloxypsoralen) system, most residues showed RMSF values of approximately 1.0–2.5 Å, with more frequent local fluctuations than those observed for CPD‐1 (Figure 8b). Although several ligand‐binding residues remained within low‐RMSF regions, the surrounding residues exhibited greater flexibility, indicating comparatively higher conformational variability around the binding region. The CPD‐3 (Cnidilin) system showed generally low residue fluctuations, with most RMSF values ranging from approximately 0.8 to 2.0 Å (Figure 8c). Most ligand‐binding residues were located in relatively stable regions, although several localized regions exhibited higher flexibility. For the CPD‐4 (Phellopterin) system, most residues displayed RMSF values of approximately 1.0–2.5 Å, with more pronounced fluctuations in several regions (Figure 8d). The key binding residues generally remained in regions of relatively low fluctuation, although the surrounding regions were more flexible than those observed for CPD‐1.

**FIGURE 8:**
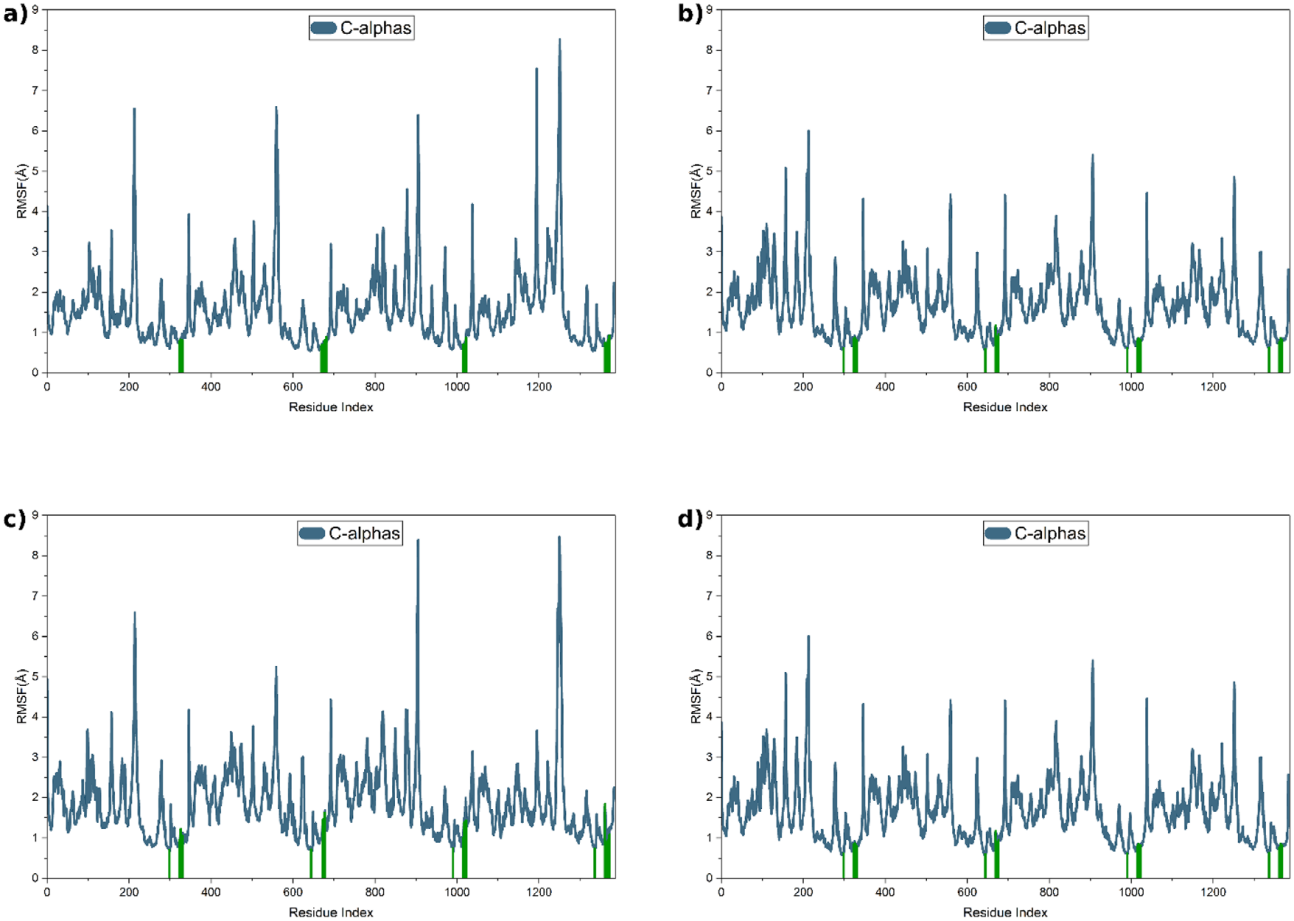
Root‐mean‐square fluctuations (RMSF) of the complexes, calculated from 200 ns molecular dynamics simulations to describe local changes in the protein chains, with residue numbers on the x‐axis and RMSF values (in Å) on the y‐axis. (a) RMSF values for the CPD‐1 (Bergamottin)–Kv1.3 channel complex; (b) RMSF values for the CPD‐2 (8‐geranyloxypsoralen)–Kv1.3 channel complex; (c) RMSF values for the CPD‐3 (Cnidilin)–Kv1.3 channel complex; (d) RMSF values for the CPD‐4 (Phellopterin)–Kv1.3 channel complex.

Overall, the RMSF profiles indicated that the ligand‐binding regions remained relatively stable in all four complexes, with CPD‐1 and CPD‐3 generally exhibiting lower local conformational variability than CPD‐2 and CPD‐4.

### 3.10 Analysis of Protein‐Ligand Interactions

Protein–ligand contact analysis of the 200 ns molecular dynamics simulations revealed distinct interaction patterns among the four Kv1.3–ligand complexes (Figure 9). CPD‐1 (Bergamottin) formed persistent hydrophobic contacts with Val469, Ile472, Ala473, and Val476, with the interaction fraction of Ile472 in subunit B exceeding 0.5; water‐bridge interactions were also observed. These features supported a stable binding mode and were consistent with its high Kv1.3 inhibition rate (96.1%).

**FIGURE 9:**
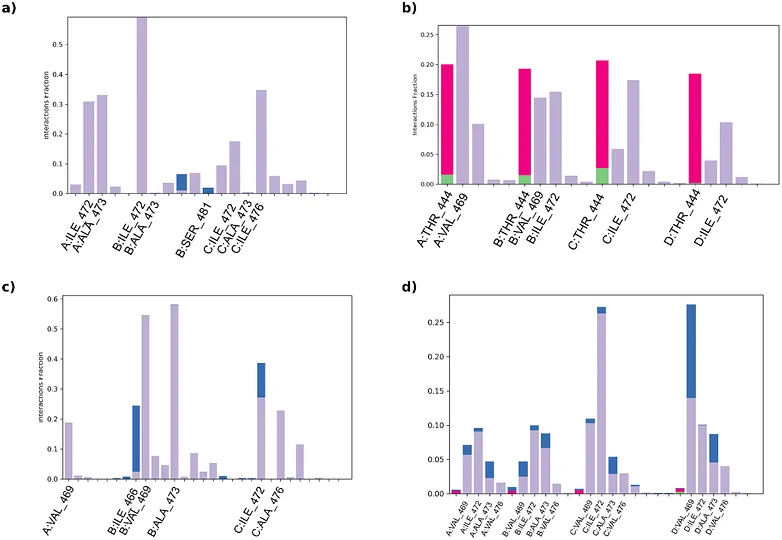
Bar charts showing protein‐ligand interactions in 200 ns molecular dynamics. (a) CPD‐1 (Bergamottin) complex with the Kv1.3 channel; (b) CPD‐2 (8‐geranyloxypsoralen) complex with the Kv1.3 channel; (c) CPD‐3 (Cnidilin) complex with the Kv1.3 channel; (d) CPD‐4 (Phellopterin) complex with the Kv1.3 channel.

CPD‐2 (8‐geranyloxypsoralen) exhibited more dispersed interactions, with most interaction fractions below 0.25 and no comparable high‐occupancy hydrophobic network, consistent with its weaker inhibitory activity (19.7%). CPD‐3 (Cnidilin) showed concentrated hydrophobic contacts with Val469, Ile472, Ala473, and Val476, with major interaction fractions of approximately 0.55, 0.58, and 0.39. This relatively persistent interaction network was consistent with its higher inhibition rate (78.7%) and IC_50_ value of 5.31 µM. In contrast, CPD‐4 (Phellopterin) displayed more dispersed contacts, with the highest interaction fraction of approximately 0.29 and most others below 0.13. Its inhibition rate was 75.7%, while its higher IC_50_ value of 9.41 µM indicated slightly weaker inhibitory potency than CPD‐3.

Overall, CPD‐1 and CPD‐3 formed more concentrated and higher‐occupancy interaction networks, whereas CPD‐2 and CPD‐4 exhibited more dispersed and less persistent contacts.

## 4 Discussion

In our study, we identified CPD‐1 and CPD‐2, as well as CPD‐3 and CPD‐4, which are C‐5‐substituted and C‐8‐substituted compounds, respectively, derived from the same skeleton. At the computational level, there is a certain trend indicating that the C‐5‐substituted compounds scored higher than the C‐8‐substituted compounds [42]. Electrophysiological evaluation showed a pronounced difference between CPD‐1 and CPD‐2, whereas CPD‐3 and CPD‐4 exhibited similar inhibition at the screening concentration; however, CPD‐3 showed a lower IC_50_ value than CPD‐4 (5.31 vs. 9.41 µM). Overall, these results indicate a trend toward greater potency for the C‐5‐substituted compounds. In the molecular dynamics simulations of CPD‐1–CPD‐4, the C‐5‐substituted compounds also showed more favorable RMSD, RMSF, and protein–ligand interaction profiles than the corresponding C‐8‐substituted compounds. Both C‐5‐substituted and C‐8‐substituted compounds possess similar hydrophobic side‐chain structures; however, due to differences in their side‐chain attachment sites, their spatial orientations differ significantly. Molecular dynamics simulation results show that C‐5‐substituted compounds can extend their side chains into the hydrophobic cavity formed by Val469 and Ile472 in an optimal orientation, making it easier for them to form sustained and stable hydrophobic interactions with these key residues, thereby exhibiting better binding stability [16]. This observation is consistent with the structure of the established Kv1.3 inhibitor PAP‐1 [43], which belongs to the class of 5‐position‐substituted compounds. Notably, this interpretation is supported by an earlier matched‐pair study in which relocation of the 4‐phenoxybutoxy side chain of PAP‐1 from C‐5 to C‐8, without further modification of the psoralen scaffold, reduced Kv1.3 inhibitory potency from approximately 2 nM to 1 µM [42]. The apparent IC_50_ value obtained here for PAP‐1 (230 nM) was higher than the 2 nM value originally reported for this compound [10]. Such discrepancies are well documented: in a systematic comparison, IC_50_ values for PAP‐1 ranging from 0.4 nM to 2.67 µM were obtained for the same compound depending on the expression system and recording method used [44]. This variability arises because PAP‐1 exhibits use‐ and state‐dependent blockade of Kv1.3, with preferential binding to the C‐type inactivated state; differences in pulse duration, depolarization voltage, stimulation frequency, and the time allowed to reach equilibrium block therefore substantially influence its apparent potency [10, 44]. The value obtained in the present study falls within this reported range. Because PAP‐1 and all eight furanocoumarins were evaluated under identical recording conditions, the relative comparisons reported here remain internally consistent, although absolute potency values should not be compared directly with those obtained under different protocols. Although CPD‐1 and CPD‐2 show clear inhibitory activity, their potencies remain in the low‐ µM range, substantially weaker than optimized synthetic blockers such as PAP‐1. Selectivity against other potassium channels, effects on T‐cell function, and pharmacokinetic properties have not yet been evaluated. Nevertheless, the observed trend favoring C‐5 substitution provides useful structure–activity insight and offers a practical starting point for the rational design of more potent furanocoumarin‐based Kv1.3 inhibitors.

## 5 Conclusion

This study employed an integrated computational–experimental strategy combining virtual screening, natural product isolation, electrophysiological validation, cytotoxicity evaluation, and molecular dynamics simulations to identify natural Kv1.3 inhibitors from the furanocoumarins of *A. dahurica*. A library of 43 reported furanocoumarins was systematically evaluated using molecular docking and MM/GBSA calculations. In parallel, major furanocoumarins were isolated and characterized from *A. dahurica*, and representative positional isomers were selected for subsequent biological validation. Comparative analysis of four matched pairs showed an overall trend favoring the C‐5‐substituted compounds, with three of the four C‐5‐substituted analogues exhibiting greater inhibitory potency than their corresponding C‐8‐substituted isomers. Among the eight compounds selected for experimental validation, CPD‐1 showed both the highest docking score (−6.51 kcal/mol) and the most favorable MM/GBSA estimate (−34.30 kcal/mol). Electrophysiological studies demonstrated that both CPD‐1 and CPD‐3 inhibited Kv1.3 currents with IC_50_ values of 5.70 µM and 5.31 µM, respectively, and showed no detectable cytotoxicity at 10 µM. Additional concentration–response analyses showed IC_50_ values of 22.42, 9.41, 7.04, 4.27, 8.26, and 19.81 µM for CPD‐2, CPD‐4, CPD‐5, CPD‐6, CPD‐7, and CPD‐8, respectively. Molecular dynamics simulations further indicated that these two compounds formed stable protein–ligand complexes, as evidenced by favorable RMSD and RMSF profiles and persistent interactions with key residues, including Thr444, Val469, Ile472, and Ala473. In contrast, the corresponding isomers CPD‐2 and CPD‐4 exhibited weaker and less stable interactions, consistent with their higher IC_50_ values. Notably, the superior performance of CPD‐1 and CPD‐3 is consistent with the structural characteristics of the well‐established Kv1.3 inhibitor PAP‐1, further supporting the importance of substitution position in determining Kv1.3 inhibitory activity. Collectively, these findings identify CPD‐1 and CPD‐3 as promising natural lead compounds for Kv1.3‐targeted drug discovery. More importantly, the observed preference for the C‐5‐substituted compounds over their C‐8 isomers provides preliminary structure–activity relationship (SAR) insight that may facilitate the rational design and optimization of furanocoumarin‐derived Kv1.3 inhibitors. Given the pivotal role of Kv1.3 in T‐cell activation and immune‐mediated inflammatory diseases, these compounds represent attractive candidates for further investigation as potential therapeutics for autoimmune and allergic inflammatory disorders. Moreover, the integrated computational–experimental workflow established in this study provides an efficient strategy for discovering bioactive ion‐channel modulators from natural products. Future studies should focus on evaluating in vivo efficacy, selectivity against other potassium channel subtypes, pharmacokinetic properties, and structural optimization to further advance the therapeutic potential of these lead compounds.

## Author Contributions

**Bingnan Han** and **Yujie Chen**: conceived the idea and designed the research. **Yueshi Sha** and Yujie Chen: completed material preparation, characterization, data analysis and manuscript writing. **Xianya Zhao** completed the characterization and data analysis. **Binglin Lei**, **Liyang Guan** and **Nabeel Khalid Bhutta** assisted in the preparation of the material. Nabeel Khalid Bhutta validated, reviewed and revised the manuscript. **Fan Zhang** and Bingnan Han, revised and approved the manuscript version.

## Conflicts of Interest

The authors declare no conflicts of interest.

## Supporting information




**Supporting File 1**: cbdv71923‐sup‐0001‐SuppMat.docx

## Data Availability

All data supporting the findings of this study are available within the article and its Supporting Information.

## References

[1] N. Chaachouay and L. Zidane , “Plant‐derived Natural Products: A Source for Drug Discovery and Development,” Drugs and Drug Candidates 3(2024): 184–207, 10.3390/ddc3010011.

[2] A. G. Atanasov , S. B. Zotchev , V. M. Dirsch , and C. T. Supuran , “Natural Products in Drug Discovery: Advances and Opportunities,” Nature reviews Drug discovery 20 (2021): 200–216, 10.1038/s41573-020-00114-z. PMC7841765 33510482

[3] D. J. Newman and G. M. Cragg , “Natural Products as Sources of New Drugs Over the Nearly Four Decades From 01/1981 to 09/2019,” Journal of Natural Products 83 (2020): 770–803, 10.1021/acs.jnatprod.9b01285. 32162523

[4] H. Zhao , Y.‐L. Feng , M. Wang , J.‐J. Wang , T. Liu , and J. Yu , “The Angelica dahurica: A Review of Traditional Uses, Phytochemistry and Pharmacology,” Frontiers in Pharmacology 13 (2022): 896637, 10.3389/fphar.2022.896637. PMC9283917 35847034

[5] H. Gao and Q. Li , “The Prediction of Antioxidant Q‐Markers for Angelica Dahurica Based on the Dynamics Change in Chemical Compositions and Network Pharmacology,” Molecules 28 (2023): 5248, 10.3390/molecules28135248. PMC10343566 37446909

[6] C. Zhu , M. Wang , J. Guo , et al., “ *Angelica dahurica* Extracts Attenuate CFA‐Induced Inflammatory Pain via TRPV1 in Mice,” Evidence‐Based Complementary and Alternative Medicine 2022 (2022): 4684830, 10.1155/2022/4684830. PMC9152374 35656472

[7] Y.‐Y. Li , Y.‐Y. Liu , X. Zeng , et al., “Complete Mitochondrial Genome of *Angelica dahurica* and Its Implications on Evolutionary Analysis of Complex Mitochondrial Genome Architecture in Apiaceae,” Frontiers in Plant Science 15 (2024): 1367299, 10.3389/fpls.2024.1367299. PMC11074370 38716337

[8] A. Teisseyre , K. Środa‐Pomianek , A. Palko‐Labuz , and M. Chmielarz , “Voltage‐Gated Potassium Channels Kv1. 3 in Health and Disease,” in Cell Physiology–Annual Volume (InTechOpen, 2023), 10.5772/intechopen.113769.

[9] M. Navarro‐Perez , J. Capera , A. Benavente‐Garcia , S. Cassinelli , M. Colomer‐Molera , and A. Felipe , “Kv1. 3 in the Spotlight for Treating Immune Diseases,” Expert Opinion on Therapeutic Targets 28 (2024): 67–82, 10.1080/14728222.2024.2315021. 38316438

[10] A. Schmitz , A. Sankaranarayanan , P. Azam , et al., “Design of PAP‐1, a Selective Small Molecule Kv1.3 Blocker, for the Suppression of Effector Memory T Cells in Autoimmune Diseases,” Molecular Pharmacology 68 (2005): 1254–1270, 10.1124/mol.105.015669. 16099841

[11] W. Nguyen , B. L. Howard , D. S. Neale , et al., “Use of Kv1.3 Blockers for Inflammatory Skin Conditions,” Current Medicinal Chemistry 17 (2010): 2882–2896, 10.2174/092986710792065072. PMC3337760 20858170

[12] P. C. Agu , C. A. Afiukwa , O. U. Orji , et al., “Molecular Docking as a Tool for the Discovery of Molecular Targets of Nutraceuticals in Diseases Management,” Scientific Reports 13 (2023): 13398, 10.1038/s41598-023-40160-2. PMC10435576 37592012

[13] A. A. Alzain , F. A. Elbadwi , T. H. Shoaib , et al., “Integrating Computational Methods Guided the Discovery of Phytochemicals as Potential Pin1 Inhibitors for Cancer: Pharmacophore Modeling, Molecular Docking, MM‐GBSA Calculations and Molecular Dynamics Studies,” Frontiers in Chemistry 12 (2024): 1339891, 10.3389/fchem.2024.1339891. PMC10839060 38318109

[14] S. Yin , Q. Hu , J. Luo , et al., “Loureirin B, an Essential Component of Sanguis Draxonis, Inhibits Kv1.3 Channel and Suppresses Cytokine Release From Jurkat T Cells,” Cell & Bioscience 4 (2014): 78, 10.1186/2045-3701-4-78. PMC4417528 25937895

[15] P. Selvakumar , A. I. Fernández‐Mariño , N. Khanra , et al., “Structures of the T Cell Potassium Channel Kv1.3 With Immunoglobulin Modulators,” Nature Communications 13 (2022): 3854, 10.1038/s41467-022-31285-5. PMC9253088 35788586

[16] S. Zhang , N. Chen , F. Wu , et al., “Discovery of Therapeutic Promising Natural Products to Target Kv1. 3 Channel, a Transmembrane Protein Regulating Immune Disorders, Through Multidimensional Virtual Screening, Molecular Dynamics Simulations and Biological Validation,” International Journal of Biological Macromolecules 308 (2025): 142636, 10.1016/j.ijbiomac.2025.142636. 40158604

[17] A. Farhangi , N. Sarı , F. Arslan , and F. Okuş , “Indole‐based Schiff Base Derivatives and Their Fe (III) Complexes as Pancreatic Lipase Inhibitors: Synthesis, *In Silico* Studies, and Molecular Docking,” Journal of Molecular Structure 1381 (2026): 147546, 10.1016/j.molstruc.2026.147546.

[18] R. A. Friesner , R. B. Murphy , M. P. Repasky , et al., “Extra Precision Glide: Docking and Scoring Incorporating a Model of Hydrophobic Enclosure for Protein‐ligand Complexes,” Journal of Medicinal Chemistry 49 (2006): 6177–6196, 10.1021/jm051256o. 17034125

[19] C. Wang , S. Wang , S. Zhai , et al., “An OpenMM‐Based ML/MM–MMGBSA Workflow for End‐point Protein–Ligand Binding Energy Ranking,” Journal of Chemical Information and Modeling 66 (2026): 10356–10368, 10.1021/acs.jcim.6c01539. 42670866

[20] N. Vivekananthan , V. Nath , C. Marimuthu , N. Kumar , C. V. Rao , and V. Selvaraj , “Structure‐Based Screening of Antiviral Phytocompounds From the IMPPAT Database Against SARS‐CoV‐2 Main Protease Using Molecular Docking, MM‐GBSA, MD Simulation, and DFT Analysis,” In Silico Research in Biomedicine 1 (2025): 100102, 10.1016/j.insi.2025.100102.

[21] G. Fan , R. Deng , L. Zhou , et al., “Development of a Rapid Resolution Liquid Chromatographic Method Combined With Chemometrics for Quality Control of Angelicae Dahuricae Radix,” Phytochemical Analysis 23 (2012): 299–307, 10.1002/pca.1358. 21960288

[22] J. Feng , C. Qin , X. Liu , et al., “Nematicidal Coumarins From Cnidium Monnieri Fruits and *Angelica dahurica* Roots and Their Physiological Effect on Pine Wood Nematode (*Bursaphelenchus xylophilus*),” Molecules 28 (2023): 4109, 10.3390/molecules28104109. PMC10223656 37241850

[23] H.‐L. Shi , Y.‐W. Jia , S.‐L. Peng , X. Liao , L.‐S. Ding , and Y.‐M. Liu , “Metabolic Study of Angelica Dahurica Extracts Using a Reusable Liver Microsomal Nanobioreactor by Liquid Chromatography–Mass Spectrometry,” Biomedical Chromatography 29 (2015): 1514–1521, 10.1002/bmc.3451. PMC4562904 25753568

[24] A. Córdova , S. Catalán , V. Carrasco , et al., “Sustainable Assessment of Ultrasound‐assisted Extraction of Anthocyanins With Bio‐based Solvents for Upgrading Grape Pomace Cabernet Sauvignon Derived From a Winemaking Process,” Ultrasonics Sonochemistry 112 (2025): 107201, 10.1016/j.ultsonch.2024.107201. PMC11718338 39705982

[25] M. Zuccolo , A. Bassoli , A. Giorgi , L. Giupponi , S. Mazzini , and G. Borgonovo , “Phytochemical Profiling of Residual Leaves From an Alpine Landrace of Globe Artichoke (*Cynara scolymus* L.),” Molecules 30 (2025): 2649, 10.3390/molecules30122649. PMC12195720 40572612

[26] H. Nath , A. Khataniar , K. K. Bania , et al., “Nano‐functionalization and Evaluation of Antimicrobial Activity of Tinospora Cordifolia Against the TolB Protein of Pseudomonas aeruginosa—An Antibacterial and Computational Study,” Frontiers in Microbiology 14 (2023): 1138106, 10.3389/fmicb.2023.1138106. PMC10126308 37113217

[27] A. M. Ayedh , A. Adhreai , J. R. S. S. Christyraj , B. V. Yesudhason , Y. A. P. Arul Soundara Rajan , and M. Bhaswant , “Comparative Ultrasonic Bath and Probe Extraction of Piperine From Piper nigrum L. Using Natural Deep Eutectic Solvents: RSM Optimization, Characterization, and in Vitro Bioactivity,” Biomolecules 15 (2025): 1631, 10.3390/biom15111631. PMC12650695 41301549

[28] A. Panya , H. Pundith , S. Thongyim , et al., “Antibiotic‐Antiapoptotic Dual Function of *Clinacanthus nutans* (Burm. f.) Lindau Leaf Extracts Against Bovine Mastitis,” Antibiotics (Basel, Switzerland) 9 (2020): 429, 10.3390/antibiotics9070429. PMC7400556 32708141

[29] M. H. Oueslati , A. Guetat , J. Bouajila , A. K. Alzahrani , and J. Basha , “ *Deverra tortuosa* (Desf.) DC From Saudi Arabia as a New Source of Marmin and Furanocoumarins Derivatives With α‐glucosidase, Antibacterial and Cytotoxic Activities,” Heliyon 7 (2021): e06656, 10.1016/j.heliyon.2021.e06656. PMC8056227 33898812

[30] J. Gębalski , M. Małkowska , S. Wnorowska , et al., “Ethyl Acetate Fraction From *Eleutherococcus divaricatus* Root Extract as a Promising Source of Compounds With Anti‐Hyaluronidase, Anti‐Tyrosinase, and Antioxidant Activity but Not Anti‐Melanoma Activity,” Molecules 29 (2024): 3640, 10.3390/molecules29153640. PMC11313944 39125044

[31] J. Dang , Y. Lv , A. A. El‐Aty , and G. Li , “Practically Complementary Size Exclusion Chromatography and Reversed‐phase Liquid Chromatography for the Preparative Separation of Structurally Similar Flavone‐C‐glycosides,” Journal of Chromatography A 1740 (2025): 465558, 10.1016/j.chroma.2024.465558. 39631126

[32] B. Slavik , S. Roehrer , H. M. Loos , M. Minceva , and A. Buettner , “Isolation of Sesquiterpenoids From Matricaria chamomilla by Means of Solvent Assisted Flavor Evaporation and Centrifugal Partition Chromatography,” Analytical and Bioanalytical Chemistry 413 (2021): 4387–4396, 10.1007/s00216-021-03400-w. PMC8245379 34050388

[33] S. Hou , Y. W. Yi , H. J. Kang , et al., “Novel Carbazole Inhibits Phospho‐STAT3 Through Induction of Protein‐tyrosine Phosphatase PTPN6,” Journal of Medicinal Chemistry 57 (2014): 6342–6353, 10.1021/jm4018042. PMC5852381 24978112

[34] L. Miller and M. Mahoney , “Evaluation of Flash Supercritical Fluid Chromatography and Alternate Sample Loading Techniques for Pharmaceutical Medicinal Chemistry Purifications,” Journal of Chromatography A 1250 (2012): 264–273, 10.1016/j.chroma.2012.06.029. 22771260

[35] E. F. Queiroz , A. Alfattani , A. Afzan , L. Marcourt , D. Guillarme , and J. L. Wolfender , “Utility of Dry Load Injection for an Efficient Natural Products Isolation at the Semi‐Preparative Chromatographic Scale,” Journal of Chromatography A 1598 (2019): 85–91, 10.1016/j.chroma.2019.03.042. 30926257

[36] Q. W. Zhang , L. G. Lin , and W. C. Ye , “Techniques for Extraction and Isolation of Natural Products: A Comprehensive Review,” Chinese medicine 13 (2018): 20, 10.1186/s13020-018-0177-x. PMC5905184 29692864

[37] T. Kowalska and M. Sajewicz , “Thin‐Layer Chromatography (TLC) in the Screening of Botanicals‐Its Versatile Potential and Selected Applications,” Molecules 27 (2022): 6607, 10.3390/molecules27196607. PMC9572063 36235143

[38] Z. Wang , K. Zan , X.‐W. Hu , et al., “The Simultaneous Determination of Nine Furocoumarins in *Angelica dahurica* Using UPLC Combined With the QAMS Approach and Novel Health Risk Assessment Based on the Toxic Equivalency Factor,” Separations 10 (2023): 508, 10.3390/separations10090508.

[39] Y. Jiang , G. Yang , and C. Wang , DNA Vaccines: Methods and Protocols (Springer, 2020), 3–12, 10.1007/978-1-4939-0410-5.

[40] P. Thomas and T. G. Smart , “HEK293 cell Line: A Vehicle for the Expression of Recombinant Proteins,” Journal of Pharmacological and Toxicological Methods 51 (2005): 187–200, 10.1016/j.vascn.2004.08.014. 15862464

[41] B. Taciak , M. Białasek , A. Braniewska , et al., “Evaluation of Phenotypic and Functional Stability of RAW 264.7 Cell Line Through Serial Passages,” PLoS ONE 13 (2018): e0198943, 10.1371/journal.pone.0198943. PMC5995401 29889899

[42] S. B. Bodendiek , C. Mahieux , W. Hänsel , and H. Wulff , “4‐Phenoxybutoxy‐substituted Heterocycles–A Structure–Activity Relationship Study of Blockers of the Lymphocyte Potassium Channel Kv1,” European Journal of Medicinal Chemistry 44 (2009): 1838–1852, 10.1016/j.ejmech.2008.10.033. PMC2662044 19056148

[43] F. Yao , Y. Zhang , Y. Zheng , et al., “The Kv1. 3 Blocker PAP‑1 Alleviates Psoriasis‑Like Dermatitis in Rats via Suppressing Inflammation and Angiogenesis,” Communications Biology (2026), 10.1038/s42003-026-10718-7. PMC13748653 42477078

[44] Š. Gubič , L. A. Hendrickx , X. Shi , et al., “Design of New Potent and Selective Thiophene‐Based KV1.3 Inhibitors and Their Potential for Anticancer Activity,” Cancers 14 (2022): 2595, 10.3390/cancers14112595. PMC9179341 35681571

