# Supplementary material for: Discovery of Natural Kv1.3 Channel Inhibitors From Furanocoumarins of Angelica dahurica via Integrative Computational Screening and Electrophysiological Validation
Source: Chem Biodivers. 2026 Nov 14;23(11):e71923. doi: 10.1002/cbdv.71923 (PMC13769036; doi:10.1002/cbdv.71923)
Supplement: Supplementary file 1 [file CBDV-23-e71923-s001.docx]

**Discovery of Natural Kv1.3 Channel Inhibitors from Furanocoumarins of *Angelica dahurica* via Integrative Computational Screening and Electrophysiological Validation**

Yujie Chen^a,1^, Yueshi Sha^a,1^, Xianya Zhao^b^, Binglin Lei^a^, Liyang Guan^a^, Nabeel Khalid Bhutta^a^, Fan Zhang* ^b^, Bingnan Han* ^a^

^a^ Zhejiang Provincial Engineering Research Center of New Technologies and Applications for Targeted Therapy of Major Diseases, Laboratory of Anti-allergy Functional Molecules, College of Life Sciences and Medicine, Zhejiang Sci-Tech University, Hangzhou 310018, China.

^b^ State Key Laboratory of Natural Medicines, School of Traditional Chinese Pharmacy, China Pharmaceutical University, Nanjing, China.

* Corresponding author: Bingnan Han. College of Life Sciences and Medicine, Zhejiang Sci-Tech University, Hangzhou, 310018, China, E-mail address: [hanbingnan@zstu.edu.cn](mailto:hanbingnan@zstu.edu.cn).

¹ These authors contributed equally to this work.

**Contents**

[**1.Results**	4](#_Toc236852464)

[**Separation and Purification of Components from n-Hexane and Dichloromethane Extracts of Angelica dahurica**	4](#_Toc236852465)

[**Fig S1.** LC-MS Mass Spectra of *Angelica dahurica* Extract in Positive and Negative Ion Modes.	4](#_Toc236852466)

[**Table S1.** The 14 major furanocoumarins identified.	4](#_Toc236852467)

[**Fig S2.** MPLC preparative chromatogram of the n-hexane extract.	5](#_Toc236852468)

[**Fig S3.** Preparative HPLC chromatograms of furanocoumarins from the n-hexane extract of *Angelica dahurica* **(a)** Isolation of Z-S1 from fraction Z2; **(b)** Isolation of Z-06, Z-07, and Z-08 from fraction Z3; (c) Isolation of Z-09 from fraction Z4; (d) Isolation of Z-10 from fraction Z5. Detection wavelength: 310 nm.	6](#_Toc236852469)

[**Fig S4.** Analytical HPLC chromatograms of furanocoumarins isolated from the n-hexane extract of *Angelica dahurica* (a) Z-S1; (b) Z-06; (c) Z-07; (d) Z-08; (e) Z-09; (f) Z-10. Detection wavelength: 310 nm.	6](#_Toc236852470)

[**Figure S5.** MPLC preparative chromatogram of the CH₂Cl₂ extract from *Angelica dahurica*.	7](#_Toc236852471)

[**Fig S6.** HPLC chromatograms of furanocoumarins from the CH₂Cl₂ extract of *Angelica dahurica* (a) Preparative HPLC chromatogram of fraction E5; (b) Analytical HPLC chromatogram of E-5-1; (c) Analytical HPLC chromatogram of E-5-2. Detection wavelength: 310 nm.	7](#_Toc236852472)

[**Fig S7.** (a) ESI⁺ mass spectrum, (b) ¹H NMR spectrum, and (c) ¹³C NMR spectrum of compound. Z-S1.	8](#_Toc236852473)

[**Table S2.** Assignment Table for the NMR Data of Compound Z-S1.	9](#_Toc236852474)

[**Fig S8.** (a) ESI⁺ mass spectrum, (b) ¹H NMR spectrum, and (c) ¹³C NMR spectrum of compound Z-06.	10](#_Toc236852475)

[**Table S3.** Assignment Table for the NMR Data of Compound Z-06.	11](#_Toc236852476)

[**Fig S9.** (a) ESI⁺ mass spectrum, (b) ¹H NMR spectrum, and (c) ¹³C NMR spectrum of compound Z-07.	12](#_Toc236852477)

[**Table S4.** Assignment Table for the NMR Data of Compound Z-07.	13](#_Toc236852478)

[**Fig S10.** (a) ESI⁺ mass spectrum, (b) ¹H NMR spectrum, and (c) ¹³C NMR spectrum of compound Z-08.	14](#_Toc236852479)

[**Table S5.** Assignment Table for the NMR Data of Compound Z-08.	15](#_Toc236852480)

[**Fig S11.** (a) ESI⁺ mass spectrum, (b) ¹H NMR spectrum, and (c) ¹³C NMR spectrum of compound Z-09.	16](#_Toc236852481)

[**Table S6.** Assignment Table for the NMR Data of Compound Z-09.	17](#_Toc236852482)

[**Fig S12.** (a) ESI⁺ mass spectrum, (b) ¹H NMR spectrum, and (c) ¹³C NMR spectrum of compound Z-10.	18](#_Toc236852483)

[**Table S7.** Assignment Table for the NMR Data of Compound Z-10.	19](#_Toc236852484)

[**Fig S13.** (a) ESI⁺ mass spectrum, (b) ¹H NMR spectrum, and (c) ¹³C NMR spectrum of compound E-5-1.	20](#_Toc236852485)

[**Table S8.** Assignment Table for the NMR Data of Compound E-5-1.	21](#_Toc236852486)

[**Fig S14.** (a) ESI⁺ mass spectrum, (b) ¹H NMR spectrum, and (c) ¹³C NMR spectrum of compound E-5-2.	22](#_Toc236852487)

[**Table S9.** Assignment Table for the NMR Data of Compound E-5-2.	23](#_Toc236852488)

[**Electrophysiological experiments to verify inhibitory activity and IC_50_ determination**	23](#_Toc236852489)

**Fig S15.** Inhibition of hKv1.3 currents by PAP-1 and the eight tested compounds at a screening concentration of 10 μM………………………....24

**Fig S16.** Fig S16. Concentration-dependent inhibition of hKv1.3 currents by CPD-1–CPD-8……………………………...……..………………………...25

[**Compound Toxicity Studies**	26](#_Toc236852491)

[**Fig S17.** (a) The effect of the compound on L929 cell viability at a concentration of 10 μM. (b) The effect of the compound on L929 cell viability at a concentration of 10 μM.	26](#_Toc236852492)

[**Molecular Dynamics Study**	26](#_Toc236852493)

[**Fig S18.** (a) Schematic diagram of the 3D protein-protein interaction between CPD-1 and Kv1.3. (b) Schematic diagram of the 3D interaction between CPD-2 and the Kv1.3 protein. (c) Schematic diagram of the 3D interaction between CPD-3 and the Kv1.3 protein. (d) Schematic diagram of the 3D interaction between CPD-4 and the Kv1.3 protein.	26](#_Toc236852494)

[**Reference**	27](#_Toc236852495)

**1.Results**

**Separation and Purification of Components from n-Hexane and Dichloromethane Extracts of *Angelica dahurica***

**Fig S1.** LC-MS Mass Spectra of *Angelica dahurica* Extract in Positive and Negative Ion Modes.

**Table S1.** The 14 major furanocoumarins identified.

| **Compound** | **Molecular formula** | **Molecular mass** | **Mass-to-Charge Ratio (m/z)** | **Adductions** |
| --- | --- | --- | --- | --- |
| Heraclenin | C16H14O5 | 286.0841 | 269.081 | [M+H-H2O]+ |
| Byakangelicol | C17H16O6 | 316.0947 | 299.0915 | [M+H-H2O]+ |
| Isopimpinellin | C13H10O5 | 246.0528 | 247.06 | [M+H]+ |
| (Rac)-Byakangelicin | C17H18O7 | 334.1053 | 317.1018 | [M+H-H2O]+ |
| Imperatorin | C16H14O4 | 270.0892 | 271.0965 | [M+H]+ |
| Phellopterin | C17H16O5 | 300.0998 | 301.107 | [M+H]+ |
| Bergapten | C12H8O4 | 216.0423 | 217.0494 | [M+H]+ |
| (-)-Oxypeucedanin hydrate | C16H16O6 | 304.0947 | 305.1017 | [M+H]+ |
| Oxypeucedanin | C16H14O5 | 286.0841 | 287.0912 | [M+H]+ |
| Isoimperatorin | C16H14O4 | 270.0892 | 271.0964 | [M+H]+ |
| Nodakenetin | C14H14O4 | 246.0892 | 245.0819 | [M-H]- |
| Smyrindioloside | C20H24O10 | 424.137 | 423.1295 | [M-H]- |
| Bergaptol | C11H6O4 | 202.0266 | 201.0193 | [M-H]- |
| Alloimperatorin | C16H14O4 | 270.0892 | 269.0817 | [M-H]- |

**Fig S2.** MPLC preparative chromatogram of the n-hexane extract from *Angelica dahurica*.

**Fig S3.** Preparative HPLC chromatograms of furanocoumarins from the n-hexane extract of *Angelica dahurica* (a) Isolation of Z-S1 from fraction Z2; (b) Isolation of Z-06, Z-07, and Z-08 from fraction Z3; (c) Isolation of Z-09 from fraction Z4; (d) Isolation of Z-10 from fraction Z5. Detection wavelength: 310 nm.

**Fig S4.** Analytical HPLC chromatograms of furanocoumarins isolated from the n-hexane extract of *Angelica dahurica* (a) Z-S1; (b) Z-06; (c) Z-07; (d) Z-08; (e) Z-09; (f) Z-10. Detection wavelength: 310 nm.

**Figure S5.** MPLC preparative chromatogram of the CH₂Cl₂ extract from *Angelica dahurica*.

**Fig S6.** HPLC chromatograms of furanocoumarins from the CH₂Cl₂ extract of *Angelica dahurica* (a) Preparative HPLC chromatogram of fraction E5; (b) Analytical HPLC chromatogram of E-5-1; (c) Analytical HPLC chromatogram of E-5-2. Detection wavelength: 310 nm.

**Fig S7.** (a) ESI⁺ mass spectrum, (b) ¹H NMR spectrum, and (c) ¹³C NMR spectrum of compound. Z-S1.

Compound Z-S1 is a white, needle-like crystal (petroleum ether) that is soluble in methanol. HRESI-MS yielded an [M+H]⁺ peak at m/z 271, confirming the molecular formula as C₁₆H₁₄O₄. UV (MeOH) λmax: 196, 204, 221, 249, 310 nm, exhibiting characteristic absorption bands of furanocoumarins.

¹H NMR (400 MHz, CDCl₃) δ: 8.15 (1H, d, J = 9.8 Hz, H-4), 7.59 (1H, d, J = 2.4 Hz, H-18), 7.29 (1H, s, H-5), 6.95 (1H, d, J = 2.4 Hz, H-17), 6.26 (1H, d, J = 9.8 Hz, H-3), 5.53 (1H, t, J = 7.0 Hz, H-13), 4.91 (2H, d, J = 7.0 Hz, H-14), 1.80 (3H, s, H-20), 1.70 (3H, s, H-11). ¹³C NMR (101 MHz, CDCl₃) δ: 161.45 (C-2), 158.21 (C-7), 152.71 (C-9), 149.04 (C-5), 139.73 (C-4), 119.17 (C-13), 69.79 (C-14), 25.94 (C-11), 18.34 (C-20), etc. The above data are essentially consistent with those reported for isoimperatorin in the literature [1]; therefore, Z-S1 was identified as isoimperatorin.

**Table S2.** Assignment Table for the NMR Data of Compound Z-S1.

| **Position** | **δH (ppm), multiplicity (J in Hz),Integration** | **δC (ppm)** |
| --- | --- | --- |
| 2 |  | 161.45 |
| 3 | 6.26 (d, J = 9.8, 1H) | 112.57 |
| 4 | 8.15 (d, J = 9.8, 1H) | 139.73 |
| 5 | 7.14 (s, 1H) | 149.04 |
| 6 |  | 114.21 |
| 7 |  | 158.21 |
| 8 |  | 94.24 |
| 9 |  | 152.71 |
| 10 |  | 107.53 |
| 11 | 1.70 (s, 3H) | 25.94 |
| 12 |  | 139.94 |
| 13 | 5.53 (t, J = 7.0, 1H) | 119.17 |
| 14 | 4.91 (d, J = 7.0, 2H) | 69.79 |
| 17 | 6.95 (d, J = 2.4, 1H) | 105.18 |
| 18 | 7.59 (d, J = 2.4, 1H) | 144.99 |
| 20 | 1.80 (s, 3H) | 18.34 |

**Fig S8.** (a) ESI⁺ mass spectrum, (b) ¹H NMR spectrum, and (c) ¹³C NMR spectrum of compound Z-06.

Compound Z-06 is a white, needle-like crystal (in methanol). HRESI-MS yields [M+H]⁺ m/z 217, with the molecular formula C₁₂H₈O₄.

UV λmax: 196, 221, 228, 248, 314 nm. ¹H NMR (400 MHz, CDCl₃) δ: 8.15 (d, J = 9.8 Hz, H-4), 7.59 (s), 7.02 (s), 6.27 (d, J = 9.8 Hz, H-3), 4.27 (s, OCH₃). ¹³C NMR δ: 162.07 (C=O), 60.01 (OCH₃). The above data are consistent with those reported for bergapten in the literature [1]; therefore, the compound was identified as bergapten.

**Table S3.** Assignment Table for the NMR Data of Compound Z-06.

| **Position** | **δH (ppm), multiplicity (J in Hz), Integration** | **δC (ppm)** |
| --- | --- | --- |
| 2 |  | 162.07 |
| 3 | 6.27 (d, J = 9.8, 1H) | 112.75 |
| 4 | 8.15 (d, J = 9.8, 1H） | 139.96 |
| 5 |  | 149.64 |
| 6 |  | 112 |
| 7 |  | 158.45 |
| 8 | 7.13 (s, 1H) | 93.65 |
| 9 |  | 152.45 |
| 10 |  | 106.3 |
| 11 | 4.27 (s, 3H) | 60.01 |
| 14 | 7.02 (dd, J = 2.4, 1H) | 105.05 |
| 15 | 7.59 (d, J = 2.4, 1H) | 144.95 |

**Fig S9.** (a) ESI⁺ mass spectrum, (b) ¹H NMR spectrum, and (c) ¹³C NMR spectrum of compound Z-07.

Z-07 is a white, flaky crystal (in methanol). HRESI-MS yielded [M+H]⁺ m/z 271, with the molecular formula C₁₆H₁₄O₄. UV λmax: 196, 217, 247, 298 nm, consistent with the characteristics of furanocoumarins.

¹H NMR (400 MHz, CDCl₃) δ: 7.75 (1H, d, J = 9.5 Hz, H-4), 7.68 (1H, d, J = 2.2 Hz, H-13), 7.35 (1H, s, H-5), 6.81 (1H, d, J = 2.2 Hz, H-12), 6.35 (1H, d, J = 9.6 Hz, H-3), 5.60 (1H, m, H-17), 5.00 (2H, d, J = 7.2 Hz, H-16), 1.73 (3H, s, H-20), 1.71 (3H, s, H-19). ¹³C NMR δ: 161.00 (C-2), 70.57 (C-16), 26.25, 18.55. These data are consistent with those reported in the literature [1], and the compound was identified as a imperatorin.

**Table S4.** Assignment Table for the NMR Data of Compound Z-07.

| **Position** | **δH (ppm), multiplicity (J in Hz), Integration** | **δC (ppm)** |
| --- | --- | --- |
| 2 |  | 161 |
| 3 | 6.35 (d, J = 9.6, 1H) | 115.07 |
| 4 | 7.75 (d, J = 9.5, 1H) | 144.2 |
| 5 | 7.35 (s, 1H) | 113.61 |
| 6 |  | 126.29 |
| 7 |  | 149 |
| 8 |  | 132.04 |
| 9 |  | 144.83 |
| 10 |  | 116.89 |
| 12 | 6.81 (d, J = 2.2, 1H) | 107.15 |
| 13 | 7.68 (d, J = 2.2, 1H) | 147.05 |
| 16 | 5.00 (d, J = 7.2, 2H) | 70.57 |
| 17 | 5.60 (ddt, J = 8.6, 7.1, 1.4, 1H) | 120.17 |
| 18 |  | 140.19 |
| 19 | 1.71 (s, 3H) | 18.55 |
| 20 | 1.73 (s, 3H) | 26.25 |

**Fig S10.** (a) ESI⁺ mass spectrum, (b) ¹H NMR spectrum, and (c) ¹³C NMR spectrum of compound Z-08.

Z-08 is a white, flaky crystal (in methanol). HRESI-MS yielded [M+H]⁺ m/z 301, with the molecular formula C₁₇H₁₆O₅. UV λmax: 196, 224, 241, 248, 266, 310 nm, consistent with the characteristics of furanocoumarins.

¹H NMR (400 MHz, CDCl₃) δ: 8.11 (1H, d, J = 9.8 Hz, H-4), 7.61 (1H, d, J = 2.3 Hz, H-19), 6.98 (1H, d, J = 2.3 Hz, H-20), 6.27 (1H, d, J = 9.8 Hz, H-3), 5.60 (1H, m, H-14), 4.84 (2H, d, J = 7.2 Hz, H-13), 4.17 (3H, s, -OCH₃), 1.73 (3H, s, H-17), 1.70 (3H, s, H-16). ¹³C NMR δ: 160.99 (C-2), 70.79 (C-13), 61.18 (-OCH₃), 26.24, 18.50. The above data are consistent with those reported for **phellopterin** in the literature [1]; therefore, the compound is identified as **phellopterin**.

**Table S5.** Assignment Table for the NMR Data of Compound Z-08.

| **Position** | **δH (ppm), multiplicity (J in Hz), Integration** | **δC (ppm)** |
| --- | --- | --- |
| 2 |  | 160.99 |
| 3 | 6.27 (d, J = 9.8, 1H) | 113.14 |
| 4 | 8.11 (d, J = 9.8 Hz, 1H) | 139.86 |
| 5 |  | 144.8 |
| 6 |  | 114.92 |
| 7 |  | 151.21 |
| 8 |  | 127.26 |
| 9 |  | 144.74 |
| 10 |  | 107.93 |
| 13 | 4.84 (d, J = 7.2, 2H) | 70.79 |
| 14 | 5.60 (ddt, J = 7.3, 5.9, 1.5, 1H) | 120.26 |
| 15 |  | 140.08 |
| 16 | 1.70 (s, 3H) | 18.5 |
| 17 | 1.73 (s, 3H) | 26.24 |
| 19 | 7.61 (d, J = 2.3, 1H) | 145.51 |
| 20 | 6.98 (d, J = 2.3, 1H) | 105.52 |
| 22 | 4.17 (s, 3H) | 61.18 |

**Fig S11.** (a) ESI⁺ mass spectrum, (b) ¹H NMR spectrum, and (c) ¹³C NMR spectrum of compound Z-09.

Z-09 is a white powder (in methanol). HRESI-MS yielded [M+H]⁺ m/z 287, with the molecular formula C₁₆H₁₆O_5_. UV λmax: 196, 211, 226, 248, 312 nm, consistent with the characteristics of furanocoumarins.

¹H NMR (400 MHz, CDCl₃) δ: 8.21 (1H, d, J = 9.8 Hz, H-4), 7.61 (1H, d, J = 2.4 Hz, H-13), 7.20 (1H, s, H-5), 6.95 (1H, d, J = 1.0 Hz, H-14), 6.32 (1H, d, J = 9.8 Hz, H-3), 4.60 (1H, dd, J = 10.8, 4.4 Hz, H-16a), 4.44 (1H, dd, J = 10.8, 6.5 Hz, H-16b), 3.37 (3H, s, -OCH₃), 3.23 (1H, m, H-17), 1.41 (3H, s, H-21), 1.33 (3H, s, H-20). ¹³C NMR δ: 161.20 (C-2), 72.45 (C-16), 61.24 (C-17), 58.49 (-OCH₃), 24.74, 19.16. The above data are consistent with those reported for oxypeucedanin in the literature[2]; therefore, the compound is identified as oxypeucedanin.

**Table S6.** Assignment Table for the NMR Data of Compound Z-09.

| **Position** | **δH (ppm), multiplicity (J in Hz), Integration** | **δC (ppm)** |
| --- | --- | --- |
| 2 |  | 161.2 |
| 3 | 6.32 (d, J = 9.8, 1H), | 113.3 |
| 4 | 8.21 (d, J = 9.8, 1H) | 139.16 |
| 5 | 7.20 (s, 1H) | 148.48 |
| 6 |  | 114.33 |
| 7 |  | 158.17 |
| 8 |  | 95.03 |
| 9 |  | 152.68 |
| 10 |  | 107.57 |
| 13 | 7.61 (d, J = 2.4, 1H) | 145.45 |
| 14 | 6.95 (d, J = 1.0, 1H), | 104.63 |
| 16 | 4.60 (dd, J = 10.8, 4.4 Hz, 1H), 4.44 (dd, J = 10.8, 6.5, 1H) | 72.45 |
| 17 | 3.23 (dd, J = 6.5, 4.4, 1H) | 61.24 |
| 19 |  | 58.49 |
| 20 | 1.33 (s, 3H) | 19.16 |
| 21 | 1.41 (s, 3H) | 24.74 |

**Fig S12.** (a) ESI⁺ mass spectrum, (b) ¹H NMR spectrum, and (c) ¹³C NMR spectrum of compound Z-10.

Z-10 is a white, needle-like crystal (in methanol). HRESI-MS yielded [M+Na]⁺ m/z 339, with the molecular formula C₁₇H₁₆O₆. UV λmax: 197, 224, 231, 250, 318 nm, consistent with the characteristics of furanocoumarins.

¹H NMR (400 MHz, CDCl₃) δ: 8.13 (1H, d, J = 9.8 Hz, H-4), 7.63 (1H, d, J = 2.4 Hz), 7.01 (1H, d, J = 2.4 Hz), 6.28 (1H, d, J = 9.8 Hz, H-3), 4.44 (2H, d, H₂-12), 4.19 (3H, s, -OCH₃), 1.33, 1.25 (3H each, s, 2×CH₃). ¹³C NMR δ: 162.3 (C-2), 61.5 (-OCH₃). The above data are consistent with those reported for byakangelicol in the literature[3]; therefore, the compound is identified as byakangelicol.

**Table S7.** Assignment Table for the NMR Data of Compound Z-10.

| **Position** | **δH (ppm), multiplicity (J in Hz), Integration** | **δC (ppm)** |
| --- | --- | --- |
| 2 |  | 160.32 |
| 3 | 6.28 (d, J = 9.7, 1H) | 112.94 |
| 4 | 8.13 (d, J = 9.8, 1H) | 139.59 |
| 5 |  | 144.31 |
| 6 |  | 114.52 |
| 7 |  | 150.66 |
| 8 |  | 126.76 |
| 9 |  | 144.99 |
| 10 |  | 107.56 |
| 12 | 4.44 (d, J = 5.6, 2H) | 72.86 |
| 13 | 3.30 (t, J = 5.6, 1H) | 61.51 |
| 14 |  | 58.19 |
| 15 | 1.25 (s, 3H) | 18.96 |
| 16 | 1.33 (s, 3H) | 24.69 |
| 19 | 4.19 (s, 3H) | 60.81 |
| 21 | 7.63 (d, J = 2.3, 1H) | 145.34 |
| 22 | 7.01 (d, J = 2.3, 1H) | 105.38 |

**Fig S13.** (a) ESI⁺ mass spectrum, (b) ¹H NMR spectrum, and (c) ¹³C NMR spectrum of compound E-5-1.

E-5-1 is a pale yellowish-white granular crystal (in methanol). HRESI-MS yielded [M+H]⁺ m/z 305, with the molecular formula C₁₆H₁₆O₆. UV λmax: 220, 249, 310 nm, consistent with the characteristics of furanocoumarins.

¹H NMR (400 MHz, CDCl₃) δ: 8.16 (1H, d, J = 9.8 Hz, H-4), 7.60 (1H, d, J = 2.4 Hz, H-13), 7.14 (1H, s, H-8), 6.99 (1H, d, J = 2.3 Hz, H-14), 6.27 (1H, d, J = 9.8 Hz, H-3), 4.54 (1H, dd, J = 9.8, 2.9 Hz, H-16a), 4.44 (1H, dd, J = 9.7, 7.9 Hz, H-16b), 3.91 (1H, dd, J = 7.8, 2.9 Hz, H-17), 1.36 (3H, s, H-22), 1.31 (3H, s, H-21). The ¹³C NMR data are consistent with those reported in the literature[4] for oxypeucedanin hydrate; therefore, the compound is identified as oxypeucedanin hydrate.

**Table S8.** Assignment Table for the NMR Data of Compound E-5-1.

| **Position** | **δH (ppm), multiplicity (J in Hz), Integration** |
| --- | --- |
| 3 | 6.27 (d, J = 9.8, 1H) |
| 4 | 8.16 (d, J = 9.8, 1H) |
| 8 | 7.14 (s, 1H) |
| 13 | 7.60 (d, J = 2.4, 1H) |
| 14 | 6.99 (d, J = 2.3, 1H) |
| 16 | 4.54 (dd, J = 9.8, 2.9, 1H), 4.44 (dd, J = 9.7, 7.9, 1H) |
| 17 | 3.91 (dd, J = 7.8, 2.9, 1H) |
| 21 | 1.31 (s, 3H) |
| 22 | 1.36 (s, 3H) |

**Fig S14.** (a) ESI⁺ mass spectrum, (b) ¹H NMR spectrum, and (c) ¹³C NMR spectrum of compound E-5-2.

E-5-2 is a pale yellow powder (methanol). HRESI-MS yielded [M+Na]⁺ m/z 357, with a molecular formula of C₁₇H₁₈O₇. UV λmax: 223, 268, 313 nm, consistent with the characteristics of furanocoumarins.

¹H NMR (400 MHz, CDCl₃) δ: 8.13 (1H, d, J = 9.8 Hz, H-4), 7.64 (1H, d, J = 2.3 Hz, H-21), 7.02 (1H, d, J = 2.3 Hz, H-22), 6.29 (1H, d, J = 9.8 Hz, H-3), 4.60 (1H, dd, J = 10.2, 2.6 Hz, H-13a), 4.26 (1H, dd, J = 10.1, 7.9 Hz, H-13b), 4.19 (3H, s, -OCH₃), 3.83 (1H, dd, J = 8.0, 2.6 Hz, H-14), 1.32 (3H, s, H-19), 1.28 (3H, s, H-18). The ¹³C NMR data are consistent with those reported in the literature[1] for byakangelicin; therefore, the compound is identified as byakangelicin.

**Table S9.** Assignment Table for the NMR Data of Compound E-5-2.

| **Position** | **δH (ppm), multiplicity (J in Hz), Integration** |
| --- | --- |
| 3 | 6.29 (d, J = 9.8, 1H) |
| 4 | 8.13 (d, J = 9.8, 1H) |
| 13 | 4.60 (dd, J = 10.2, 2.6, 1H), 4.26 (dd, J = 10.1, 7.9, 1H) |
| 14 | 3.83 (dd, J = 8.0, 2.6, 1H) |
| 18 | 1.28 (s, 3H) |
| 19 | 1.32 (s, 3H) |
| 21 | 7.64 (d, J = 2.3, 1H) |
| 22 | 7.02 (d, J = 2.3, 1H) |
| 24 | 4.19 (s, 3H) |

**Electrophysiological experiments to verify inhibitory activity and IC50 determination**

**Fig S15.** **Inhibition of hKv1.3 currents by PAP-1 and the eight tested compounds at a screening concentration of 10 μM.** Whole-cell patch-clamp recordings were used to evaluate the inhibition of hKv1.3 currents by PAP-1 and CPD-1–CPD-8. Data are presented as the mean ± SEM from five independently recorded cells (n = 5), and error bars represent SEM. These single-concentration measurements were used for initial screening and were not interpreted as direct measures of potency. No inferential statistical comparisons were performed; therefore, P values and significance symbols are not applicable.

**Fig S16.** Concentration-dependent inhibition of hKv1.3 currents by CPD-1–CPD-8. Representative whole-cell current traces and concentration–response curves are shown for (a) CPD-1 (bergamottin), (b) CPD-2 (8-geranyloxypsoralen), (c) CPD-3 (cnidilin), (d) CPD-4 (phellopterin), (e) CPD-5 (isoimperatorin), (f) CPD-6 (imperatorin), (g) CPD-7 (oxypeucedanin), and (h) CPD-8 (oxyimperatorin). The corresponding IC₅₀ values were 5.70, 22.42, 5.31, 9.41, 7.04, 4.27, 8.26, and 19.81 μM, respectively. Data are presented as mean ± SEM (n = 5 cells per concentration), with error bars representing SEM. IC₅₀ values were obtained by nonlinear regression. No inferential statistical comparisons were performed; therefore, P values and significance symbols are not applicable.

**Compound Toxicity Studies**

**Fig S17. Cytotoxicity of CPD-1, CPD-3, and CPD-4 in L929 and RAW 264.7 cells.** (a) Cell viability of L929 mouse fibroblast cells after treatment with CPD-1, CPD-3, and CPD-4 at 10 μM for 24 h. (b) Cell viability of RAW 264.7 mouse macrophage cells after treatment with the same compounds at 10 μM for 24 h. Cisplatin (PDD, 10 μM) was used as the positive control. For both panels, data are presented as the mean ± SD from three independent experiments (n = 3), and error bars represent SD.

**Fig S18.** (a) Schematic diagram of the 3D protein-ligand interaction between CPD-1 and Kv1.3. (b) Schematic diagram of the 3D interaction between CPD-2 and the Kv1.3 protein. (c) Schematic diagram of the 3D interaction between CPD-3 and the Kv1.3 protein. (d) Schematic diagram of the 3D interaction between CPD-4 and the Kv1.3 protein.

**Reference**

[1]	Wen, J., Shi, F., Yang, W., et al., “Coumarins from of Angelicae Dahuricae Radix,” Chinese Traditional and Herbal Drugs 55, no. 19 (2024): 6465–6473, https://doi.org/10.7501/j.issn.0253-2670.2024.19.002

[2]	Mottaghipisheh, J., "Oxypeucedanin: Chemotaxonomy, Isolation, and Bioactivities," *Plants (Basel)* 10, no. 8 (2021), <https://doi.org/10.3390/plants10081577>.

[3]	Kiyonga, A. N., Hong, G., Kim, H. S., Suh, Y. G., Jung, K., "Facile and Rapid Isolation of Oxypeucedanin Hydrate and Byakangelicin from Angelica dahurica by Using [Bmim]Tf(2)N Ionic Liquid," *Molecules (Basel, Switzerland)* 26, no. 4 (2021), <https://doi.org/10.3390/molecules26040830>.

[4]	Piao, X. L., Park, I. H., Baek, S. H., Kim, H. Y., Park, M. K., Park, J. H., "Antioxidative activity of furanocoumarins isolated from Angelicae dahuricae," *Journal of ethnopharmacology* 93, no. 2-3 (2004), <https://doi.org/10.1016/j.jep.2004.03.054>.
